# Diversity of bradyrhizobial T3SS systems and their roles in symbiosis with peanut (*Arachis hypogaea*) and *Vigna* species (*V. radiata* and *V. mungo*)

**DOI:** 10.1128/aem.00600-25

**Published:** 2025-08-08

**Authors:** Tarnee Phimphong, Shun Hashimoto, Pongpan Songwattana, Jenjira Wongdee, Teerana Greetatorn, Kamonluck Teamtisong, Pakpoom Boonchuen, Sachiko Masuda, Arisa Shibata, Ken Shirasu, Phoutthasone Sibounnavong, Panlada Tittabutr, Nantakorn Boonkerd, Shusei Sato, Djamel Gully, Eric Giraud, Pongdet Piromyou, Neung Teaumroong

**Affiliations:** 1 School of Biotechnology, Institute of Agricultural Technology, Suranaree University of Technology 506974, Nakhon Ratchasima, Thailand; 2 Faculty of Agriculture, National University of Lao PDR, Vientiane, Laos; 3 Graduate School of Life Sciences, Tohoku University Symbiosis Genomics Lab, Sendai, Japan; 4 Institute of Research and Development, Suranaree University of Technology 65162, Nakhon Ratchasima, Nakhon Ratchasima, Thailand; 5 The Center for Scientific and Technological Equipment, Suranaree University of Technology 65162, Nakhon Ratchasima, Nakhon Ratchasima, Thailand; 6 RIKEN-TRIP, RIKEN Center for Sustainable Resource Science 98319, Yokohama, Kanagawa, Japan; 7 PHIM Plant Health Institute of Montpellier, Université Montpellier, IRD, CIRAD, INRAE, Institut Agro, Montpellier, France; The University of Tennessee Knoxville, Knoxville, Tennessee, USA

**Keywords:** T3SS, symbiosis, bradyrhizobial diversity, *Vigna* sp., *Arachis hypogaea*

## Abstract

Symbiosis between *Bradyrhizobium* strains isolated from Lao People’s Democratic Republic (Lao PDR) and intercropped legumes (*Arachis hypogaea*, *Vigna radiata*, and *V. mungo*) was regulated by the type III secretion system (T3SS), which delivers effector proteins (T3Es) into host plant cells to modulate nodulation. To explore this mechanism, we sequenced and analyzed seven *Bradyrhizobium* genomes, identifying putative T3Es across five T3SS groups (G.1–G.5), which were classified based on the sequence of *rhcN*, a conserved ATPase gene essential for T3SS function. Phylogenetic analysis of *rhcN* more closely reflected the evolutionary relationships of nodulation genes than those based on 16S rRNA or whole-genome comparisons, underscoring its symbiotic relevance. Functional assays using *rhcN* mutants revealed group-specific effects on nodulation; G.1 strains showed neutral effects on *A. hypogaea*, negative effects on *V. radiata*, and positive effects on *V. mungo*. G.2 strains consistently promoted nodulation across all hosts and lacked effectors related to SUMO (small ubiquitin-like modifier) pathways, which have been implicated in host defense regulation. G.3 strains reduced nodulation in *A. hypogaea* but enhanced it in *Vigna* species. G.4 strains suppressed nodulation in *A. hypogaea*, and G.5 strains inhibited nodulation across all tested legumes. These findings highlight the diversity in T3SS organization, effector composition, and symbiotic responses among native *Bradyrhizobium* strains. The identification of known and uncharacterized effectors suggests roles in host compatibility and specificity. These strains, along with their effector profiles, provide a foundation for future functional studies to better understand T3SS-mediated interactions and support the development of targeted inoculants for legume hosts.

**IMPORTANCE:**

This study advances our understanding of legume–*Bradyrhizobium* symbiosis by examining the genetic organization and evolutionary patterns of T3SS genes. Our findings revealed that T3SS gene evolution does not always align with phylogenies based on 16S rRNA or whole-genome sequences, suggesting that horizontal gene transfer and functional adaptation may shape diversification. The observed variation in T3SS architecture and effector profiles among the five distinct *Bradyrhizobium* groups was correlated with host-specific nodulation outcomes in *A. hypogaea*, *V. radiata*, and *V. mungo*. We also identified novel candidate genes influencing symbiotic signaling and compatibility. These insights into the diversity and function of T3SS components contribute to a broader understanding of host–microbe communication and may support the development of more targeted and efficient rhizobial inoculants for sustainable legume cultivation and improved biological nitrogen fixation.

## INTRODUCTION

*Rhizobium* forms mutualistic relationships with leguminous plants by stimulating the plants to develop nodule structures that enable the utilization of atmospheric di-nitrogen (N_2_) through bacterial nitrogenase. This symbiotic interaction is initiated by the specific recognition of signals between partners. Legumes produce flavonoids, which act as key signaling molecules recognized by specific *Rhizobium* species (1). These flavonoids interact with the rhizobial NodD regulator, activating nodulation (*nod*) genes and leading to the production of Nod factors (NFs) (2). Recent comparative genetics and genomics of rhizobial strains have shown that host plant nodulation is determined not only by rhizobial NFs but also by the secretion of type III secretion system (T3SS) effectors, known as T3Es (3, 4). These T3Es, commonly referred to as nodulation outer proteins (Nops), are directly transferred into eukaryotic host cells (3). T3SS synthesis is naturally triggered by plant flavonoids through the rhizobial NodD transcriptional regulator system, which subsequently induces T3SS transcription regulator (*ttsI*) expression, which activates the expression of T3SS apparatus genes and T3Es (5). Previous research has shown that some rhizobial T3Es play different roles depending on their interactions with host plant proteins, as several *Bradyrhizobium* strains deliver a mixture of T3Es into host cells, interfering with cellular processes to suppress the host immune system and promote mutualistic symbiosis (6–10). In contrast, some T3Es stimulate plant defense reactions, thereby triggering incompatibility for nodulation symbiosis (11–13). Based on these reports, T3Es are one of the crucial factors influencing the effectiveness of *Bradyrhizobium*–legume symbiosis.

Various *Bradyrhizobium* species, including *B. arachidis*, *B. lablabi*, *B. ferriligni, B. embrapense*, *B. pachyrhizi*, *B. elkanii*, and *B. guangxisense*, have been isolated directly from the root nodules of peanuts (*Arachis hypogaea*) (14–16). In Lao PDR, *A. hypogaea* is the major bean produced, and it is often co-cultivated with other legumes, such as *Vigna radiata* and *V. mungo*, to enhance soil fertility through biological nitrogen fixation (17). In a recent study by Phimphong (17), several isolated *Bradyrhizobium* strains from Lao PDR significantly promoted *A. hypogaea* growth, and some of the *Bradyrhizobium* strains enhanced mutualistic symbiosis in *Vigna*; certain *Bradyrhizobium* strains inhibited nodule formation in *Vigna* species. The mechanisms regulating such host-dependent symbiotic interactions with these legumes remain unclear. However, these differences in the efficiency between the strains and the co-cultivated crop species may be related to the T3SS, which can positively or negatively modulate the symbiotic interaction. Although the role of T3SS in *Bradyrhizobium* strains native to the Lao PDR that interact with these leguminous plants has not yet been elucidated (17, 18), the specific interactions of *A. hypogaea* and *Vigna* species with *Bradyrhizobium* T3SSs remain largely unexplored. Investigating the diversity of T3SSs in these indigenous *Bradyrhizobium* strains offers a unique opportunity for understanding their influence on the symbiosis with *A. hypogaea* and *Vigna*. This exploration may also reveal novel T3Es that regulate interactions between *Bradyrhizobium* T3SSs and these leguminous hosts, thus providing valuable insights into the symbiotic mechanisms involved.

## RESULTS

### Phylogenetic analyses and gene organization and comparison of the T3SSs

**Fig 1:**
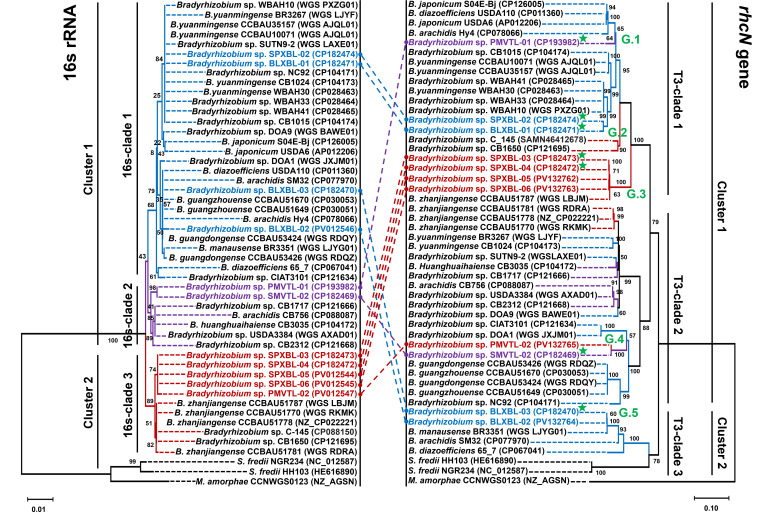
Phylogenetic tree comparing the 16S rRNA and *rhcN* genes from 50 rhizobial strains. This phylogenetic tree illustrates the evolutionary relationships between the 16S rRNA and *rhcN* genes in *Bradyrhizobium* and related species. Color coding represents bradyrhizobia isolated from Lao PDR, grouped into different clades: blue for 16s-clade 1, purple for 16s-clade 2, red for 16s-clade 3, and green (G.1–G.5) for groups separated based on the *rhcN* gene tree. Strains with a whole-genome sequence in this study are marked with a green star. The tree was constructed using the maximum likelihood (ML) method, applying the Kimura 2-parameter (K2P) model with a Gamma distribution (+G) and five rate categories. Bootstrap analysis with 1,000 replicates was performed to evaluate the statistical support of the branches. The scale bar represents 0.01% and 0.10% sequence divergence, reflecting the genetic differences among the analyzed strains.

In this study, we analyzed 11 *Bradyrhizobium* strains isolated from *A. hypogaea* nodules. These strains, including strains PMVTL-01, PMVTL-02, SPXBL-02, SPXBL-03, SPXBL-04, SPXBL-05, SPXBL-06, SMVTL-02, BLXBL-02, and BLXBL-03, exhibited variations in nodulation efficiency on *A. hypogaea* and *Vigna* species (17). We conducted a comparative phylogenetic analysis of the 16S rRNA and T3SS (*rhcN*) genes, focusing on their evolutionary relationships. The *rhcN* gene encodes a conserved ATPase component of the T3SS. To achieve this, we included related species obtained from public databases, such as MaGe and NCBI. The results revealed that the 16S rRNA gene sequences formed three distinct clades, which were grouped into two major clusters (Fig. 1). In cluster 1, *Bradyrhizobium* sp. BLXBL-01 and SPXBL-02 were closely related to undescribed *Bradyrhizobium* species from India (WBAH10, WBAH33), Thailand (SUTN9-2), and Bolivia (NC92) and *B. yuanmingense* strains from China and India. Additionally, *Bradyrhizobium* sp. BLXBL-02 and BLXBL-03 were grouped with *B. diazoefficiens* USDA110 (USA), *B. guangzhouense* CCBAU51670 and CCBAU51649 (China), and *B. arachidis* Hy4 and SM32 (China). In clade 2, *Bradyrhizobium* sp. PMVTL-01 and SMVTL-02 were closely related and clustered together with *Bradyrhizobium* sp. CB1717 (Brazil), *B. arachidis* CB756 (Zimbabwe), and *B. huanghuaihaiense* CB3035 (China). In cluster 2, five *Bradyrhizobium* strains, SPXBL-03 to SPXBL-06 and PMVTL-02, formed a distinct group that was slightly separated from other *Bradyrhizobium* strains in the phylogenetic tree (16S-clade 3).

Similarly, phylogenetic analysis of the *rhcN* gene revealed three distinct clades, which were grouped into two major clusters (Fig. 1). In cluster 1 (T3-clade 1), strains SPXBL-02 and BLXBL-01 were grouped with *Bradyrhizobium* sp. WBAH10 and WBAH33, displaying a similar evolutionary pattern to the 16S rRNA gene (16S-clade 1). Interestingly, strain PMVTL-01 was positioned in T3-clade 1 for the *rhcN* gene despite being placed in 16S-clade 2 based on the 16S rRNA gene. Additionally, strains SPXBL-03 to SPXBL-06 formed a distinct phylogenetic subgroup within the *rhcN* group (T3-clade 1) and were slightly separated from other *Bradyrhizobium* strains, which belonged to 16s-clade 3 based on the 16S rRNA gene. Phylogenetic incongruences were particularly evident in strains SMVTL-02 and PMVTL-02, which clustered together based on *rhcN* (T3-clade 2) but were placed in different 16s clades (SMVTL-02 in 16s-clade 2 and PMVTL-02 in 16s-clade 3), suggesting the shared origin of their *rhcN* genes despite differing genomic backgrounds. Based on the *rhcN* phylogenetic tree, the *Bradyrhizobium* strains isolated from Lao PDR were classified into five groups: PMVTL-01 (G.1), BLXBL-01 and SPXBL-02 (G.2), SPXBL-03 to SPXBL-06 (G.3), PMVTL-02 and SMVTL-02 (G.4), and BLXBL-02 and BLXBL-03 (G.5). The division into these five groups was specific to the *rhcN* gene analysis conducted in this study and did not represent a general classification of *rhcN* sequences. Notably, the two strains in group 4 were phylogenetically distant based on their 16S rRNA gene, while the other strains within the same *rhcN* cluster were also grouped based on their 16S sequences. This discordance highlights that the evolution of the *rhcN* gene does not always correspond to that of the 16S rRNA gene (19).

### Phylogenetic distribution of *nod*, *nif*, and T3SS genes

**Fig 2:**
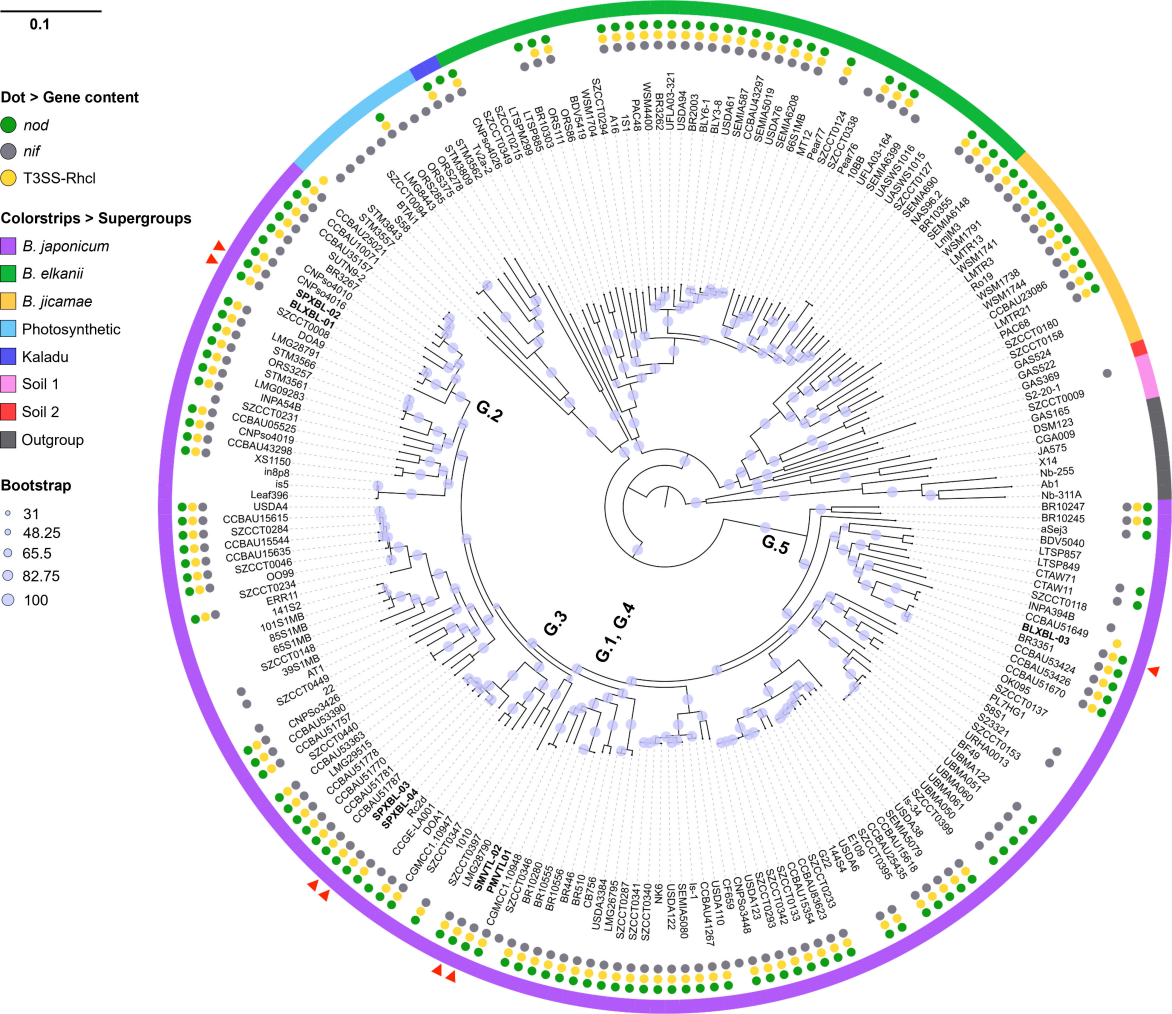
Phylogenomic tree of *Bradyrhizobium* strains based on the alignment of 718 core genes shared by at least 98% of the strains. Red arrowheads indicate the seven newly sequenced strains in this study (groups G.1–G.5). Colored dots represent gene presence: green = *nod*, gray = *nif*, yellow = T3SS-*rhcI*. Outer color strips indicate supergroups based on phylogenetic clustering. The tree was inferred using ModelFinder, with branch support estimated by ultrafast bootstrap (100,000 iterations). Scale bar represents 0.1% sequence divergence. Isolation substrates/hosts: our strains and *Bradyrhizobium* sp. WBAH10, WBAH30, WBAH33, WBAH41, and *B. zanjianense* CCBAU51787 – *A. hypogaea; B. diazoefficiens* USDA110, *B. japonicum* USDA6, and *B. cosmicum* 58S1 – *G. max; Bradyrhizobium* sp. DOA9, DOA1, and SUTN9-2 – *A. americana; B. manausense* BR3351 – *V. unguiculata; B. iriomotense* SZCCT0346 – soil; and *B. cosmicum* S23321 – paddy field soil.

To investigate the genes involved in the T3SS system, representative *Bradyrhizobium* strains from groups G.1 to G.5 were selected for whole-genome sequencing and analysis. The selected strains included PMVTL-01 (G.1), SPXBL-02 and BLXBL-01 (G.2), SPXBL-03 and SPXBL-04 (G.3), SMVTL-02 (G.4), and BLXBL-03 (G.5) (Fig. S1A through G). Genome sequencing was performed using PacBio Revio HiFi technology, and assemblies were generated using the SMRT Link platform (Table S4). Phylogenetic analysis of the resulting whole-genome sequences revealed that the *Bradyrhizobium* strains were classified into seven phylogenetic clades previously identified within the genus (20), including the *B. japonicum*, *Photosynthetic*, Kakadu, *B. elkanii*, *B. jicamae*, Soil 1, and Soil 2 clades (Fig. 2). Notably, all seven *Bradyrhizobium* strains from the Lao PDR were grouped into the *B. japonicum* clade (Fig. 2), indicating a consistent evolutionary relationship within this lineage. Whole-genome phylogeny showed that PMVTL-01 (G.1) clustered closely with SMVTL-02 (G.4), reflecting similar symbiotic traits. Most strains closely related to this group contained the *nod*, *nif*, and T3SS genes, except for strain SZCCT0346, which lacked both the *nod* and T3SS genes. Comparative genomic analysis showed that the strains in group G.2 (SPXBL-02 and BLXBL-01) clustered closely with strain SZCCT0008, which lacked genes associated with symbiosis (*nod*, *nif*, and T3SS) (Fig. 2). G.3, comprising strains SPXBL-03 and SPXBL-04, formed a distinct cluster, with closely related strains also containing the *nod*, *nif*, and T3SS genes. Similarly, G.5 (BLXBL-03) harbored these genes, whereas some closely related strains, such as CCBAU51649, lacked *nod* and *nif* genes. Additionally, strain INPA394B possessed only an atypical *nif* gene. These findings indicate that the evolution of T3SS among the tested strains was not directly correlated with whole-genome or 16S rRNA gene phylogenies (Fig. 1 and 2). Furthermore, symbiosis-related genes (*nod*, *nif*, and T3SS) within the *B. japonicum* clade exhibited symbiotic gene diversity; most strains contained all three gene types, while some lacked one or more. This variation suggests that the evolution of symbiotic traits within the *B. japonicum* clade may have been shaped by the specific host associations of each strain.

**Fig 3:**
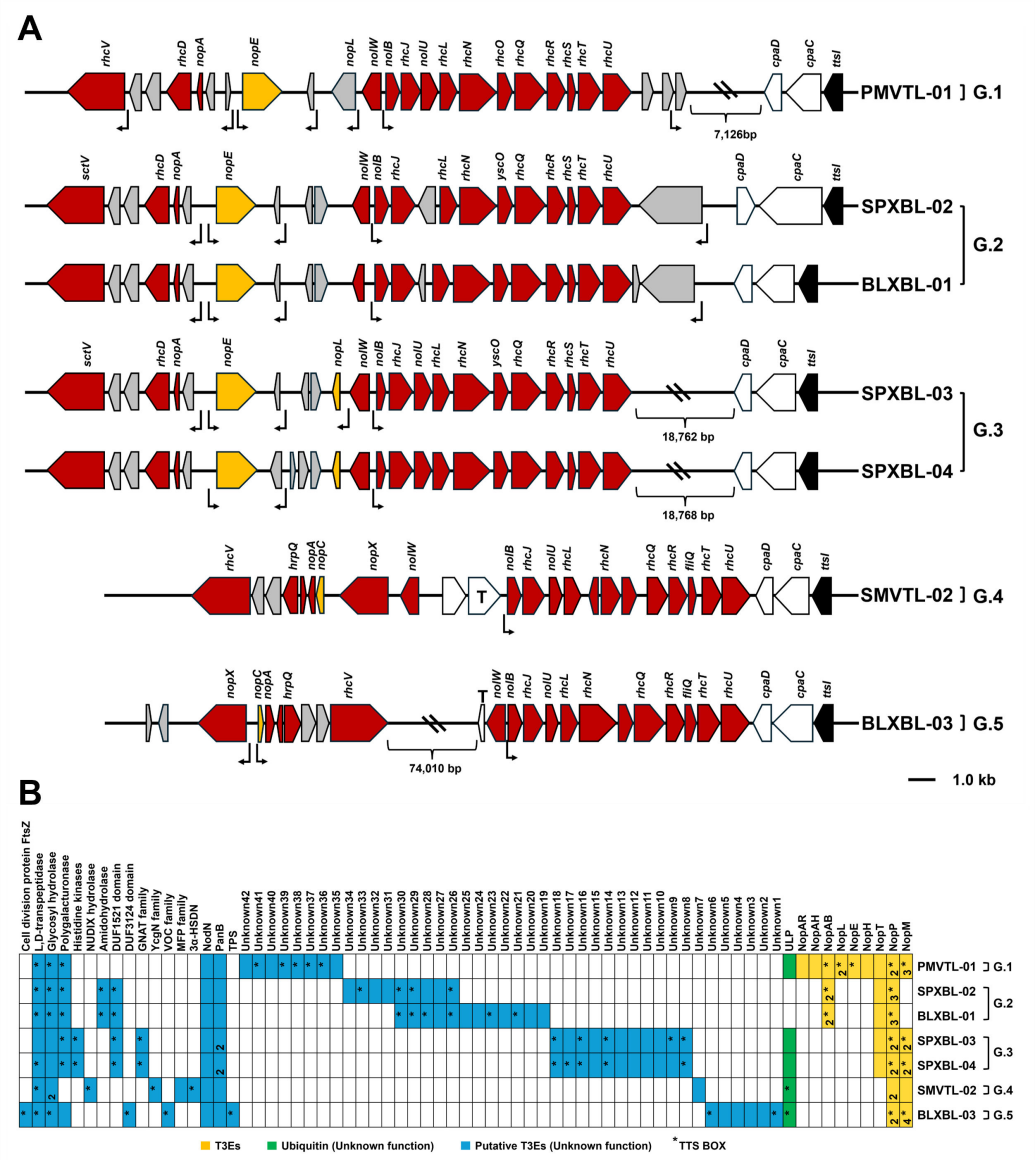
Genetic organization of the type III secretion system (T3SS) cluster and putative effectors in *Bradyrhizobium* strains (G.1–G.5). (**A**) The genetic organization of the T3SS cluster and its associated putative effectors in seven *Bradyrhizobium* strains is shown. Open reading frames (ORFs) are represented by arrows, with their orientations and sizes determined using SnapGene Viewer. The location and orientation of regulatory motifs are indicated: rectangular open arrows for *tts*-box motifs, a black rectangular arrow for the *nod*-box motif, a red rectangular arrow for the T3SS syringe, and yellow rectangular arrows for T3SS effectors (T3Es). (**B**) Prediction of T3Es and putative effectors was conducted using multiple tools, including *tts*-box detection (Python script), Effectidor analysis, and BLAST searches against a database of known effectors. Color scheme: yellow for T3Es, green for ubiquitin/SUMO-related proteins (unknown function), and blue for putative effectors (unknown function). Stars indicate the presence of *tts*-box motifs.

Whole-genome analyses were conducted to examine the organization and distribution of genes associated with symbiotic adaptation. For instance, the symbiosis island among our *Bragyrhizobium* strains ranged from the 120 to 650 kb region with a relatively low GC content (63%–64%) (Fig. S1 and Table S1). These regions also harbored multiple insertion sequences (IS elements) and integrase genes, consistent with the presence of symbiosis islands. The T3SS gene cluster, including the full *rhc* operon and the transcriptional regulator *ttsI*, was detected in all five phylogenetic groups (G.1–G.5) (Fig. 3A). For each strain, the surrounding genomic context was characterized by a low GC content, the presence of IS elements, and altered codon usage, which was consistent with horizontal acquisition of these loci (Fig. S1).

The phylogenetic distribution of the *nodABC* genes verified the clustering observed for *rhcN*, a T3SS ATPase. Strains PMVTL-01 (G.1), SPXBL-02 and BLXBL-01 (G.2), and SPXBL-03 and SPXBL-04 (G.3) were grouped into *nodABC* cluster 1, consistent with their assignment to *rhcN* cluster 1 (Fig. S2). In contrast, BLXBL-03 (G.5) was assigned to *nodABC* cluster 2, matching its classification in *rhcN* cluster 2 (Fig. 1; Fig. S2). These clustering patterns suggest a congruent distribution of nodulation and T3SS genes across *Bradyrhizobium* strains isolated from *A. hypogaea* and *Vigna* species.

Average nucleotide identity (ANI)-based comparisons of the symbiosis islands indicated that strains BLXBL-03 (G.5) and SMVTL-02 (G.4) shared similar ANIm values, while G.1–G.3 strains were similar to each other but distinct from G.4 and G.5 (Fig. S3A through C). This pattern was congruent with phylogenetic analyses of *rhcN* and *nodABC*. Specifically, G.1–G.3 strains (PMVTL-01, SPXBL-02, BLXBL-01, SPXBL-03, and SPXBL-04) grouped into the same *nodABC* and *rhcN* clusters (cluster 1), whereas BLXBL-03 (G.5) grouped into *nodABC* cluster 2 and *rhcN* cluster 2 (Fig. 1; Fig. S2). These data reflect group-specific variations in the composition of symbiosis islands across strains.

### Gene organization and comparison of T3SS genes

The T3SS genes in *Bradyrhizobium* were generally classified into three gene clusters (19). An important distinction among the analyzed strains was the type of T3SS gene cluster they possessed. Specifically, strains from groups G.4 and G.5 harbored a T3SS gene cluster of the RhcIa type, while strains from groups G.1, G.2, and G.3 contained a T3SS gene cluster of the RhcIb type. The key difference between these two types was the translocon component: NopX was associated with rhcIb, whereas NopE was associated with rhcIa (Fig. 3A). This distinction suggests that the evolution and function of the T3SS apparatus may differ among these groups. Regarding the genetic organization of *Bradyrhizobium* T3SS gene clusters, the main putative operon is preceded by a *tts*-box, a conserved cis-regulatory sequence associated with the activation of T3SS genes. In this study, we identified putative *tts*-boxes using the consensus sequence “GTCAGnTnnnnGnnAGnnnnnnnnnnnA,” as previously described (21). The presence of *tts-*box motifs upstream of the T3SS operons was used to support operon prediction, and the results are summarized in Table S5. Genes encoding secretion machinery components were identified in the following order: *rhcV*, *nopB*, *rhcJ*, *nolU*, *rhcL*, *rhcN*, *rhcO*, *rhcQ*, *rhcR*, *rhcS*, *rhcT*, and *rhcU*. Although these strains shared a similar overall T3SS gene arrangement, subtle differences were observed in the regions separating the three gene clusters constituting T3SS. Furthermore, the arrangement of injectisome genes within *Bradyrhizobium* was consistent within each group (G.1–G.3), with some intergroup variation. Interestingly, the injectisome gene arrangement in groups G.4 (SMVTL-02) and G.5 (BLXBL-03) differed from the three previously mentioned groups, reflecting the differences between the rhcIa and rhcIb clusters. Although the T3SS gene clusters of *Bradyrhizobium* shared core structural features, differences in cluster types and gene arrangements correlated with variations in symbiotic interactions. Thus, the organization of the T3SS genes was consistent with the evolutionary phylogenetic tree of the *rhcN* gene (Fig. 1 and 3A).

To identify putative effectors that may be translocated by this T3SS machinery among the seven genomes of our isolated strains, we combined three *in silico* searches: (i) identification of upstream *tts*-box promoter sequences; (ii) similarity-based screening with Effectidor; and (iii) BLASTP (Fig. 3B; Tables S1 and S5). This T3E is particularly interesting because some, such as Bel2-5 and Sup3, have been reported to induce nodule organogenesis, and others, such as NopD and, in some cases, Bel2-5, have been shown to completely inhibit nodulation (20, 22). After excluding proteins predicted to be components of the secretion apparatus (such as NopA, NopX, and NolB) (23), in group G.1 (PMVTL-01), we identified 23 putative translocated effectors, including 10 previously characterized T3Es, one SUMO-related protein, and 12 uncharacterized proteins (Fig. 3B). The SUMO (small ubiquitin-like modifier)-related protein is of particular interest due to its potential involvement in subverting host post-translational regulation pathways. Group G.2 (SPXBL-02 and BLXBL-01) displayed variability in effector composition. SPXBL-02 harbored 19 candidate effectors, and BLXBL-01 contained 22. Notably, 11 effectors differed between the two strains: SPXBL-02 uniquely carried Unknown31–Unknown34, while BLXBL-01 possessed Unknown19–Unknown25. G.2 was the only group in which no SUMO-related proteins were detected. In G.3 (SPXBL-03), 23 candidate effectors were conserved across both strains, including three known T3Es, one SUMO-related protein (ULP), and 19 uncharacterized proteins. The SUMO-related gene was validated using domain predictions from InterProScan (24). This suggests that the effector repertoire of G.3 may play a distinct role in host interactions. In G.4 (SMVTL-02), a comparatively smaller set of 13 candidate effectors was identified, comprising three known T3Es, one SUMO-related protein, and nine uncharacterized proteins. Among these, five effectors (Unknown7, 3α-HSDN, MFP family protein, YcgN family protein, and NUDIX hydrolase) were unique to this group and were not observed in others. Group G.5 (BLXBL-03) harbored 18 candidate effectors, which included several quiche proteins, such as Unknown7, cell division protein FtsZ, DUF3124 domain-containing protein, VOC family protein, and trehalose-6-phosphate synthase (TPS). Although their functions remained undefined, several genes were adjacent to *tts*-box motifs, which are regulatory sequences typically associated with T3SS effector genes. Many of these proteins have not been reported in other rhizobial species, suggesting potential novelty and specialization.

Overall, our comparative analysis of T3SS groups G.1–G.5 revealed a broader diversity of candidate T3Es than previously classified, including novel group-specific proteins and SUMO-related effectors. These findings indicate that effector variation among T3SS groups contributes to differences in symbiotic compatibility or efficiency with host legumes. To explore this hypothesis, nodulation efficiency assays comparing wild-type (WT) and T3SS injectisome mutant strains were performed.

### The T3SS plays a contrasting role in the symbiotic properties of *Bradyrhizobium* strains interacting with *Arachis hypogaea*

**Fig 4:**
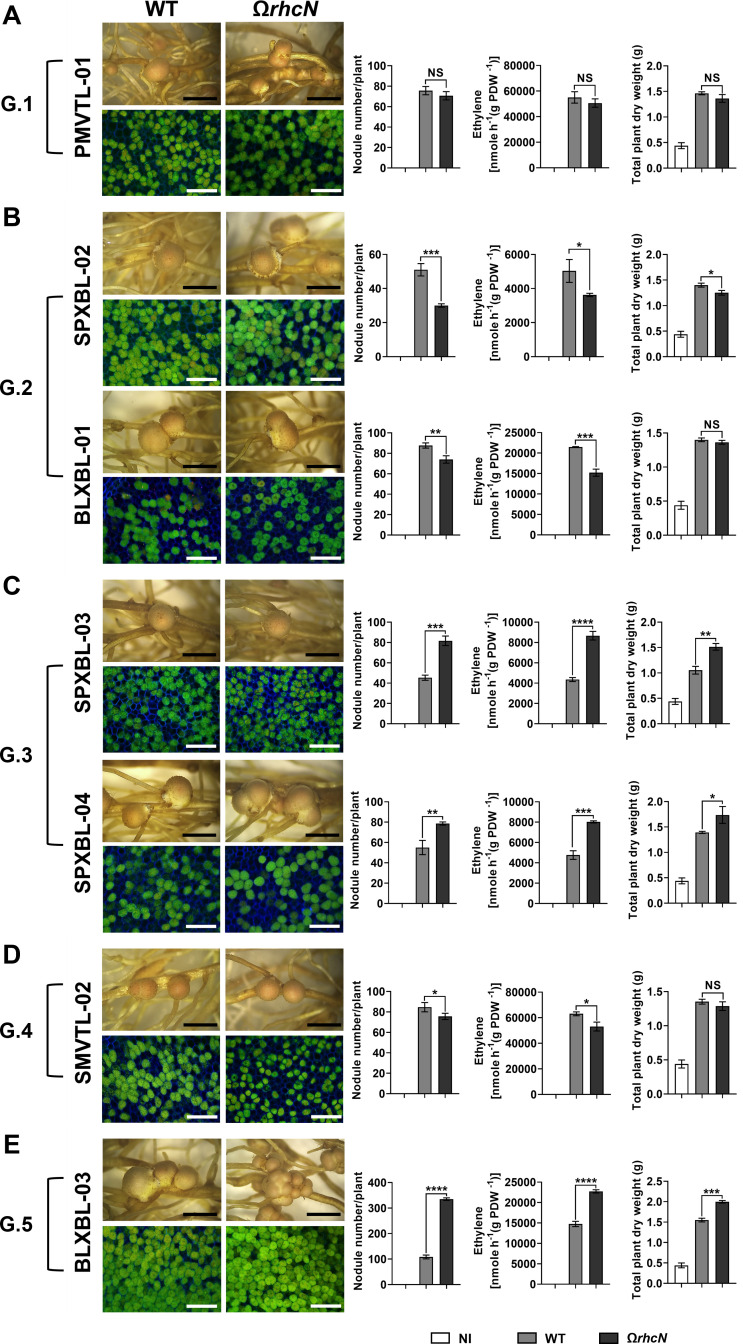
Symbiotic phenotypes of seven *Bradyrhizobium* sp. strains with *A. hypogaea*. Symbiotic phenotypes were assessed at 30 days post-inoculation (dpi) in wild-type strains (WT), their corresponding Ω*rhcN* mutants, and a non-inoculated (NI) control. (**A**) G1: PMVTL-01. (**B**) G2: SPXBL-02 and BLXBL-01. (**C**) G3: SPXBL-03 and SPXBL-04. (**D**) G4: SMVTL-02. (**E**) G5: BLXBL-03. Bacteroides in nodules were analyzed using confocal microscopy with propidium iodide (red; infected plant nuclei, dead bacteria), SYTO9 (green; live bacteria), and calcofluor-white (blue; plant cell walls). Nodule count, nitrogen fixation (ARA), and total plant dry weight were measured at 30 dpi. Scale bars: black = 1 mm, white = 100 µm (20×). Values represent the mean ± SD (*n* = 3). *t*-test significance: ns = *P* > 0.05, *P ≤ 0.05, **P ≤ 0.01, ***P ≤ 0.001, ****P ≤ 0.0001.

Since the *Bradyrhizobium* strains were previously isolated from *A. hypogaea* root nodules and their symbiotic efficiency with this plant varied significantly (17), we hypothesized that T3SSs play a crucial role in *Bradyrhizobium–A. hypogaea* interactions. To investigate the function of T3SS in the original *A. hypogaea* host, T3SS-deficient (Ω*rhcN*) strains from groups G.1–G.5 were constructed based on the *rhcN* phylogenetic tree analysis, and their symbiotic properties were analyzed in comparison to the WT. In group G.1, no effect of the *rhcN* mutation was observed, as the PMVTL-01Ω*rhcN* mutant displayed a similar symbiotic efficiency to the WT strain. Furthermore, nodule morphologies did not differ between PMVTL-01Ω*rhcN* and PMVTL-01WT inoculations, and bacteroid cells were still alive inside *A. hypogaea* nodules (Fig. 4A). The presence of T3SS itself did not directly contribute to symbiosis with *A. hypogaea*. Instead, the potential impact may be associated with specific T3Es delivered by the system, suggesting that differences in effector profiles could play a role. In G.2, inoculation with SPXBL-02WT and BLXBL-01WT resulted in significantly higher nodule formation and nitrogen fixation compared to their T3SS-deficient mutants (Fig. 4B). Regarding bacteroid morphology, no effect of the T3SS mutation was observed on bacteroid cell viability, as both the WT and Ω*rhcN* strains remained viable. This indicates that T3SS plays a crucial beneficial role (nodule formation and nitrogenase activity) in the symbiotic efficiency of these strains during symbiosis with *A. hypogaea*. In G.3, the four T3SS-deficient mutant strains tested (SPXBL-03Ω*rhcN*, SPXBL-04Ω*rhcN*, SPXBL-05Ω*rhcN*, and SPXBL-06Ω*rhcN*) exhibited significantly higher symbiotic efficiency (nodule number, nitrogen fixation, and total plant dry weight) compared to the WT strains (Fig. 4C; Fig. S4A). These results indicate that the negative impact on the symbiotic properties of these four strains with *A. hypogaea* is associated with specific T3Es rather than the T3SS itself, suggesting that differences in effector profiles result in the observed symbiotic outcomes. In G.4, T3SS had a more moderate but overall positive impact on *A. hypogaea* symbiosis. SMVTL-02*ΩrhcN* exhibited a slightly lower number of nodules and nitrogen fixation compared to its WT strain (Fig. 4D). Similarly, nodule morphologies did not differ between SMVTL-02Ω*rhcN* and SMVTL-02WT inoculations, and bacteroid cells were still alive inside *A. hypogaea* nodules (Fig. 4D). In addition, T3SS had no effect on PMVTL-02 (Fig. S4B), as PMVTL-02Ω*rhcN* exhibited a symbiotic phenotype similar to that observed with PMVTL-02WT inoculation. In G.5, the T3SS significantly enhanced symbiotic efficiency in the BLXBL-03 strain, as indicated by increased nodule formation, nitrogen fixation, and total plant dry weight observed in the plants inoculated with BLXBL-03Ω*rhcN* compared to those inoculated with WT (BLXBL-03) (Fig. 4E). In the BLXBL02 strain, T3SS played a more limited role, as more nodules were observed in the BLXBL-02Ω*rhcN* mutant compared to the WT strain, but no significant difference was observed at the level of nitrogen fixation and total plant dry weight between the mutant and the WT strain. These findings highlight that the impact of T3SS on symbiotic efficiency among *Bradyrhizobium* strains may vary depending on the specific T3Es secreted rather than the presence of T3SS itself. This reflects the diverse symbiotic phenotypes of *Bradyrhizobium* on *A. hypogaea*, as previously reported by Phimphong et al. (18).

### Role of T3SS in bradyrhizobia during their interaction with *Vigna mungo* and *V. radiata*

**Fig 5:**
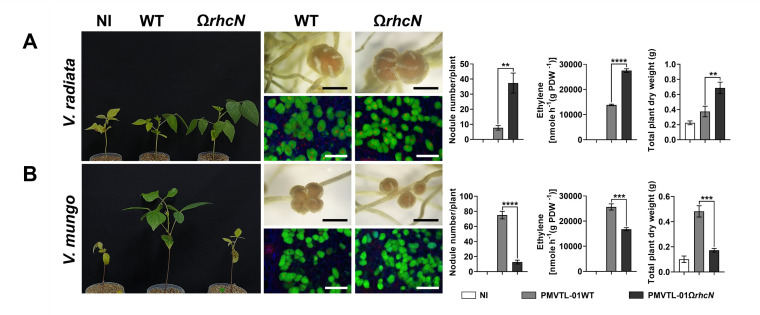
Symbiotic phenotypes of G1: PMVTL-01 with *V. radiata* (**A**) and *V. mungo* (**B**). Symbiotic phenotypes were assessed at 30 days post-inoculation (dpi) in wild-type strains (WT), their corresponding Ω*rhcN* mutants, and a non-inoculated (NI) control. Bacteroides in nodules analyzed using confocal microscopy with propidium iodide (red; infected plant nuclei, dead bacteria), SYTO9 (green; live bacteria), and calcofluor-white (blue; plant cell walls). Nodule number, nitrogen fixation (ARA), and total plant dry weight were measured at 30 dpi. Scale bars: black = 1 mm, white = 100 µm (20×). Values represent the mean ± SD (*n* = 3). *t*-test significance: ns = *P* > 0.05, *P ≤ 0.05, **P ≤ 0.01, ***P ≤ 0.001, ****P ≤ 0.0001.

**Fig 6:**
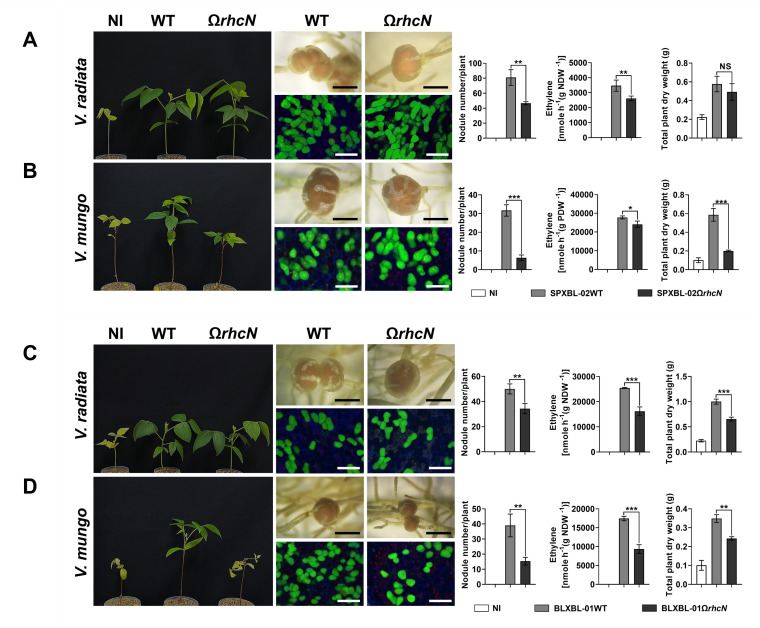
Symbiotic phenotypes of G2: SPXBL-02 (**A and B**) and BLXBL-01 (**C and D**) with *V. radiata* and *V. mungo*. Symbiotic phenotypes were assessed at 30 days post-inoculation (dpi) in wild-type strains (WT), their corresponding Ω*rhcN* mutants, and a non-inoculated (NI) control. Bacteroides in nodules were analyzed using confocal microscopy with propidium iodide (red; infected plant nuclei, dead bacteria), SYTO9 (green; live bacteria), and calcofluor-white (blue; plant cell walls). Nodule number, nitrogen fixation (ARA), and total plant dry weight were measured at 30 dpi. Scale bars: black = 1 mm, white = 100 µm (20×). Values represent the mean ± SD (*n* = 3). *t*-test significance: ns = *P* > 0.05, *P ≤ 0.05, **P ≤ 0.01, ***P ≤ 0.001, ****P ≤ 0.0001.

**Fig 7:**
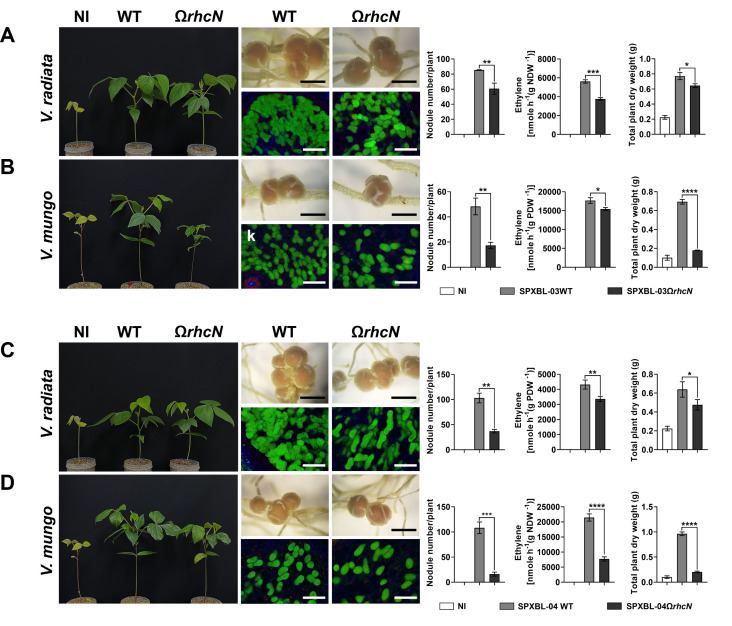
Symbiotic phenotypes of G3: SPXBL-03 (**A and B**) and SPXBL-04 (**C and D**) with *V. radiata* and *V. mungo*. Symbiotic phenotypes were assessed at 30 days post-inoculation (dpi) in wild-type strains (WT), their corresponding Ω*rhcN* mutants, and a non-inoculated (NI) control. Bacteroides in nodules were analyzed using confocal microscopy with propidium iodide (red; infected plant nuclei, dead bacteria), SYTO9 (green; live bacteria), and calcofluor-white (blue; plant cell walls). Nodule number, nitrogen fixation (ARA), and total plant dry weight were measured at 30 dpi. Scale bars: black = 1 mm, white = 100 µm (20×). Values represent the mean ± SD (*n* = 3). *t*-test significance: ns = *P* > 0.05, *P ≤ 0.05, **P ≤ 0.01, ***P ≤ 0.001, ****P ≤ 0.0001.

**Fig 8:**
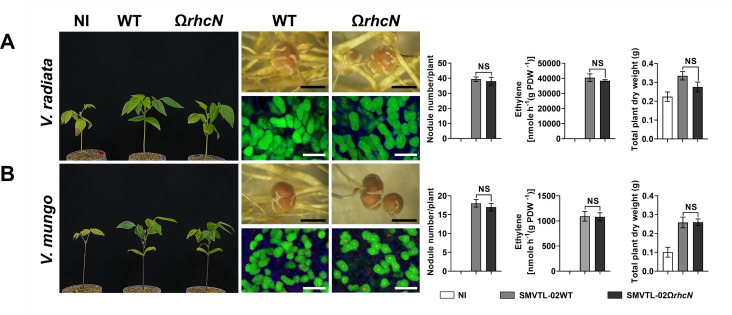
Symbiotic phenotypes of G4: SMVTL-02 with *V. radiata* (**A**) and *V. mungo* (**B**). Symbiotic phenotypes were assessed at 30 days post-inoculation (dpi) in wild-type strains (WT), their corresponding Ω*rhcN* mutants, and a non-inoculated (NI) control. Bacteroides in nodules were analyzed using confocal microscopy with propidium iodide (red; infected plant nuclei, dead bacteria), SYTO9 (green; live bacteria), and calcofluor-white (blue; plant cell walls). Nodule number, nitrogen fixation (ARA), and total plant dry weight were measured at 30 dpi. Scale bars: black = 1 mm, white = 100 µm (20×). Values represent the mean ± SD (*n* = 3). *t*-test significance: ns = *P* > 0.05, *P ≤ 0.05, **P ≤ 0.01, ***P ≤ 0.001, ****P ≤ 0.0001.

**Fig 9:**
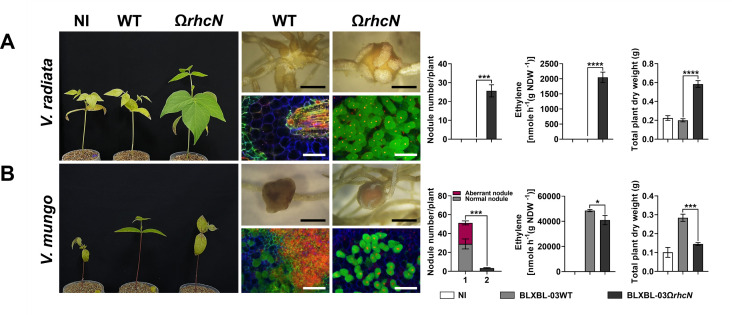
Symbiotic phenotypes of G5: BLXBL-03 with *V. radiata* (**A**) and *V. mungo* (**B**). Symbiotic phenotypes were assessed at 30 days post-inoculation (dpi) in wild-type strains (WT), their corresponding Ω*rhcN* mutants, and a non-inoculated (NI) control. Bacteroides in nodules were analyzed using confocal microscopy with propidium iodide (red; infected plant nuclei, dead bacteria), SYTO9 (green; live bacteria), and calcofluor-white (blue; plant cell walls). Nodule number, nitrogen fixation (ARA), and total plant dry weight were measured at 30 dpi. Scale bars: black = 1 mm, white = 100 µm (20×). Values represent the mean ± SD (*n* = 3). *t*-test significance: ns = *P* > 0.05, *P ≤ 0.05, **P ≤ 0.01, ***P ≤ 0.001, ****P ≤ 0.0001.

These *Bradyrhizobium* strains were isolated from an area where agricultural practices involve an integrated farming approach, including the cultivation of leguminous crops and crop rotation with *Vigna* species, such as *V. radiata* and *V. mungo*. The *Bradyrhizobium* strains may have undergone evolutionary adaptations in their T3SS genes in response to these *Vigna* species. Therefore, to better understand the role of T3SS in symbiosis with *Vigna*, we conducted experiments to compare the symbiotic efficiency between the WT and T3SS-deficient (Ω*rhcN*) strains. For G.1, T3SS negatively affected nodule formation in *V. radiata*. Specifically, the number of root nodules was significantly higher after PMVTL-01Ω*rhcN* inoculation compared to PMVTL-01WT inoculation (Fig. 5A). Moreover, the trends in nitrogen fixation and plant dry weight aligned with the results for nodule formation, with PMVTL-01Ω*rhcN* showing significantly higher values than PMVTL-01WT. In contrast, T3SS had a positive effect on symbiosis with *V. mungo*, as the number of nodules, nitrogen fixation, and plant dry weight were significantly lower after PMVTL-01Ω*rhcN* inoculation compared to PMVTL-01WT inoculation (Fig. 5B). For G.2 (SPXBL-02 and BLXBL-01) (Fig. 6) and G.3 (SPXBL-03, SPXBL-04, SPXBL-05, and SPXBL-06) (Fig. 7; Fig. S5A), T3SS had a positive effect on symbiosis with both *V. radiata* and *V. mungo*. The number of root nodules, nitrogen fixation, and plant dry weight were significantly lower in plants inoculated with *ΩrhcN* strains compared to those inoculated with WT (Fig. 6 and 7; Fig. S5A). For G.4, T3SS had no effect on symbiosis with either *V. radiata* or *V. mungo*. The number of root nodules, nitrogen fixation, and plant dry weight did not differ significantly between the WT (SMVTL-02WT and PMVTL-02WT) and T3SS mutant strains (Fig. 8; Fig. S5B). For G.5, T3SS had a strongly negative effect on nodule formation in *V. radiata* (Fig. 9; Fig. S5C). BLXBL-03Ω*rhcN* and BLXBL-02Ω*rhcN* formed more than 20 nodules per plant, whereas the WT strains (BLXBL-03WT and BLXBL-02WT) did not induce any nodules (Fig. 9; Fig. S5A). T3SS mutant strains (BLXBL-03Ω*rhcN* and BLXBL-02Ω*rhcN*) showed higher nitrogen fixation efficiency and significantly increased plant biomass compared to the WT strains (BLXBL-03WT and BLXBL-02WT), which did not form nodules (Fig. 9A; Fig. S5C). These findings indicate that T3SS negatively impacted the symbiotic association between BLXBL-03 and *V. radiata*. However, when evaluating the symbiotic efficiency of BLXBL-03 in *V. mungo*, BLXBL-03WT formed significantly more nodules than BLXBL-03Ω*rhcN*, which formed only one to two nodules per plant (Fig. 9B). Nitrogen fixation efficiency and plant dry weight analysis revealed that BLXBL-03WT exhibited significantly higher nitrogen fixation and plant growth promotion than BLXBL-03Ω*rhcN*. Thus, T3SS had a positive effect on the symbiotic properties of *V. mungo*. Although BLXBL-03WT induced a high number of nodules, some senescent nodules were observed, and approximately half of the bacteroid cells within all nodules were dead (Fig. 9B). These results indicate that although T3SS had a considerable overall positive effect on nodulation, a negative effect on bacteroid morphology was also observed. It is, therefore, possible that in the cocktail of T3Es secreted by T3SS in BLXBL-03, some play a positive role, while others play a negative role in symbiosis with *V. mungo*. The nodulation results for the three legume species revealed that the *Bradyrhizobium* strains in G.1 and G.5 exhibited a positive effect on *V. mungo* through their T3SSs, whereas T3SS had a negative impact on nodulation in *V. radiata*. In contrast, the T3SS in G.4 appeared to have no effect on symbiosis with either *V. radiata* or *V. mungo*. In G.2, T3SS exhibited a positive effect on all three tested legume species (*A. hypogaea*, *V. radiata,* and *V. mungo*).

## DISCUSSION

Legume–rhizobium symbioses are regulated by both plant host and rhizobial factors, and they are not always successful or efficient. For instance, certain bradyrhizobial type III effectors (T3Es), such as the SUMO protease (p0903) from *Bradyrhizobium* sp. DOA9 has been shown to negatively affect nodulation in *Arachis hypogaea* (13), while other effectors, such as *B. diazoefficiens* USDA110 (NopE) and *B. elkanii* USDA61 (NopP2, NopL, Bel2-5, and InnB), impact *Vigna* hosts differently depending on the cultivar (6, 10, 25–27). Because these bradyrhizobial strains were not native to *A. hypogaea* or *Vigna,* and to enhance the possibility of elucidating the role of T3SS in interactions with these legumes, we analyzed the T3SS of strains directly isolated from regions where *A. hypogaea* and *Vigna* are commonly cultivated. When considering the evolutionary comparison between the 16S rRNA gene and the T3SS injectisome-related gene (*rhcN*) among 11 isolated strains (Fig. 1), a component of the T3SS revealed both congruent and incongruent relationships among strains. For example, although PMVTL-01 and SMVTL-02 were closely related based on 16S rRNA, their *rhcN* genes differed. Conversely, the G.2 strains (BLXBL-01 and SPXBL-02) showed consistent clustering in both 16S rRNA and *rhcN* phylogenetic trees, similar to the pattern observed in the G.3 strains (SPXBL-03 and SPXBL-04). The *rhcN* genes of both groups were closely related to those of *A. hypogaea*-nodulating strain WBAH41 (28) and *B. zanjianense* CCBAU51787 (29), suggesting a possible shared ancestry. For instance, the *rhcN* gene of PMVTL-01 was similar to that of *B. diazoefficiens* USDA110 (26) and *B. japonicum* USDA6 (30), and PMVTL-01 was also phylogenetically related to the soil-isolated *B. iriomotense* SZCCT0346 (Fig. 2), a free-living, *nif*-carrying strain (31). Additionally, whole-genome phylogenetic analysis showed that USDA6 was closely related to *B. cosmicum* 58S1 and *B. cosmicum* S23321 (Fig. 2). Strain 58S1 lacked *nod* gene clusters but carried the *nif* gene. Strain S23321, isolated from paddy field soil (32), is non-nodulating but contains both the *nif* cluster and the *fixAB* and *fixCX* genes. In addition, the injectisome-related gene organization of *Bradyrhizobium* strains involved in *A. hypogaea* nodulation also showed diverse patterns (Fig. 3A). These findings imply that symbiotic genes in *Bradyrhizobium* have evolved in response to its ecological lifestyle. In G.4, strain SMVTL-02 shared *rhcN* similarity with *Bradyrhizobium* sp. DOA1, which was isolated from *Aeschynomene americana* (33), placed them in T3-clade 2 alongside *Bradyrhizobium* sp. SUTN9-2 and DOA9 (33) (Fig. 1), both of which also originated from the same host. Interestingly, the T3SS of DOA9 negatively affects nodulation in *V. radiata*, whereas the T3SS of SUTN9-2 has no effect (34), confirming that these strains may share a common origin for *rhcN* (34), but have undergone divergent evolution of their T3Es, leading to different symbiotic interactions with their legume hosts. Strains in G.5, such as BLXBL-03, clustered with *B. manausense* BR3351 (35), a strain associated with *V. unguiculata,* in the *rhcN* phylogenetic tree (Fig. 1). Additionally, the organization of injectisome-related genes in this group differed from those in the other groups, suggesting a distinct evolutionary T3SS among these strains (Fig. 3A). Interestingly, we observed partial differences in the arrangement of T3SS-related genes and the localization of the *tts*-box within the T3SS clusters (Fig. 3A), particularly in G.2 and G.3. However, when considering the evolutionary relationships based on both 16S rRNA and *rhcN* genes, we found that the 16S rRNA gene of G.2 belonged to 16S-clade 1, while G.3 was placed in 16S-clade 3, indicating distinct evolutionary lineages. In contrast, the *rhcN* genes of both groups were closely related in T3-clade 1. Based on the phylogenetic tree and the organization of injectisome-related genes, these findings suggest that these bradyrhizobial strains share a common T3SS origin, which has subsequently evolved independently within different bacterial lineages. If this scenario is true, then it could be hypothesized that horizontal gene transfer (HGT) may play an important role in bradyrhizobial T3SS evolution, as has been reported in other bacterial species, including *Pseudomonas syringae*, *Ralstonia solanacearum,* and *Salmonella enterica* (36–38). Evidence supporting HGT includes the presence of symbiosis islands containing T3SS and *nod* genes that exhibit features such as lower GC content (58–59%) compared to the whole genome (63–64%), and the presence of insertion elements associated with genomic mobility (Fig. S1; Table S1) (26, 30). This pattern also aligns with *nodABC* phylogenies, in which G.1–G.3 strains grouped in cluster 1 (with *rhcN* cluster 1), while G.4 and G.5 belonged to cluster 2 (Fig. 1; Fig. S2). ANI analysis also indicated that G.1–G.3 strains shared more similar symbiosis islands, whereas G.4 and G.5 were more distinct (Fig. S3), which could help explain differences in legume compatibility or suppression.

Our findings suggest that the effects of T3SS on symbiosis are influenced by both the bacterial strain and the host plant. In the G.1 and G.5 strains, T3SS was associated with reduced nodulation in *V. radiata*, but positive effects were observed in *V. mungo*. These outcomes indicate host-specific responses to T3Es. Such patterns may have resulted from evolutionary processes driven by agricultural practices, in which repeated association with particular legume species could have influenced effector composition and function (12, 30).

Although the core T3SS structure, including *rhc* genes and the regulator *ttsI*, was determined, variation in effector content and regulatory sequences, such as *tts*-box motifs, likely contributed to functional differences. For example, *innB* in PMVTL-01 is associated with a *tts*-box and may influence host specificity. This gene has been linked to incompatibility with *V. radiata* (10) but positively influences *V. mungo* nodulation (25), consistent with our findings. However, the absence of *innB* in *B. diazoefficiens* USDA110, a strain compatible with *V. radiata* (6), suggests that *innB* is not the only determinant of compatibility with *V. radiata*. Other T3Es likely contribute as well, further supporting the idea of host-specific roles for T3Es.

Effector profiles varied among the strain groups. SUMO-protease domain-containing T3Es were present in G.1, G.3, G.4, and G.5, but not in G.2, which was more effective on *A. hypogaea*. These effectors have also been found in pathogenic bacteria (13, 39–41), supporting their potential roles in host interaction. Moreover, in previous findings, SUMO-targeting T3Es, such as NopD and p0903, suppress nodulation by manipulating host SUMOylation pathways and triggering immune responses (13, 22). In addition to plant-driven selection, competition among co-occurring *Bradyrhizobium* strains can also influence T3SS evolution. Certain effectors may confer an advantage by affecting host signaling or suppressing nodulation by other strains (25). Thus, both host–microbe interactions and competition among microbes may contribute to shaping the diversity and function of T3SS effectors.

Overall, our data indicates that variation in T3E composition may be associated with differences in nodulation ability. G.1–G.3 strains, which were more compatible with *Vigna* species, contained effectors, such as NopE, NopT, NopAM, and NopP (Fig. 3B), which have been previously reported to promote nodulation (6, 10, 11). In contrast, G.5 strain BLXBL-03, which was less effective with *Vigna* species, lacked some of these T3Es and carried effectors potentially involved in triggering host defense responses. Furthermore, we identified many putative T3Es with unknown functions that have not yet been characterized (Fig. 3B). These findings highlight the importance of further investigating the roles of these putative T3Es in influencing the efficiency of mutualistic interactions with host plants.

In conclusion, our study revealed considerable diversity in the T3SS and effector profiles of *Bradyrhizobium* strains from new genomes associated with *A. hypogaea* and *Vigna* species. Phylogenetic comparisons of the 16S rRNA, *rhcN*, and symbiosis islands suggest that T3SS components have been shaped by both vertical inheritance and HGT. Although core T3SS genes are widely conserved, differences in effector composition, including several newly identified putative T3Es, were correlated with variations in nodulation efficiency and host specificity.

This illustrates the relationship between the presence or absence of putative T3Es—including novel candidates identified through genome analysis—and host range. Certain strains promoted nodulation in *V. mungo* but showed reduced effectiveness in *V. radiata*, indicating host-dependent roles for these proteins (Table 1). These findings suggest that the studied strains offer a promising model for future functional studies aimed at characterizing novel or uncharacterized effectors. Further validation of these putative T3Es will be essential for understanding their roles in symbiosis and for improving the selection of effective *Bradyrhizobium* inoculants.

**TABLE 1:** Phenotypic effects of wild-type (WT) bradyrhizobial strains compared with T3SS-deficient (Ω/∆T3SS) mutants on three legume hosts, along with their putative effector content^*a*^^,^^*b*^

Test strains vs. ref. strains	Putative effector content	Host range			Ref.
		Hypogaea	*V. radiata*	V*. mungo*	
		WT vs Ω/∆T3SS	WT vs Ω/∆T3SS	WT vs Ω/∆T3SS	
G.1 PMVTL-01	NopM, NopP, NopT, NopH, NopE, NopL, NopAB, NopAH, NopAR, ULP and 13 unknown	N	-	+	This study
G.2 SPXBL-02	NopP, NopT, NopAB and 16 unknown	+	+	+	This study
G.2 BLXBL-01	NopP, NopT, NopAB and 19 unknown	+	+	+	This study
G.3 SPXBL-03	NopM, NopP, NopT, ULP and 19 unknown	-	+	+	This study
G.3 SPXBL-04	NopM, NopP, NopT, ULP and 19 unknown	-	+	+	This study
G.4 SMVTL-02	NopM, NopP, ULP and 9 unknown	+	N	N	This study
G.5 BLXBL-03	NopM, NopP, ULP and 15 unknown	-	-	-	This study
USDA61	NopP2 InnB Bel2-5	NR NR NR	+ - N	+ + +	(10, 27, 42, 43)
ORS3257	NopP NopT NopAB	NR NR NR	- N N	+ + +	(11)
USDA110	NopE NopP	NR NR	+ +	NR NR	(6, 42)
DOA9	p0903	-	NR	NR	(13)

^*a*^ +, positive effect; −, negative effect; N, no effect; NR, no report; G, group.

^*b*^ The data include novel strains from this study, as well as reference strains from previous studies, including USDA61 (10, 27, 42, 43), ORS3257 (11), USDA110 (6, 42), and DOA9 (13).

## MATERIALS AND METHODS

### Bacterial strains and culture conditions

*Bradyrhizobium* strains used in this study were cultured in arabinose-gluconate (AG) medium (44) or yeast-mannitol (YM) medium (45) at 28°C in a rotary shaker at 180 rpm for four days. *Escherichia coli* strains were grown in Luria-Bertani (LB) medium (46) at 37°C for 18 h. The bacteria and plasmids used are summarized in Table S2, and the antibiotic concentrations that were added to the media were as follows: 20 µg/mL cefotaxime (cefo), 20 µg/mL nalidixic acid (Nal), 20 µg/mL gentamicin (Genta), and 100 µg/mL kanamycin (Km).

### 16S rRNA, *rhcN* gene sequencing, whole-genome sequencing, and prediction of T3E repertoires

*Bradyrhizobium* strains were cultivated in AG medium, and genomic DNA was extracted using the Bacteria Genomic Prep Mini Spin Kit (Cytiva, Marlborough, MA, USA). Amplification of the 16S rRNA gene was performed by polymerase chain reaction (PCR) using universal primer pairs fD1 and rP2 (47), and the *rhcN* gene was amplified by degenerated primer pairs rhcN.univ.f2 and rhcN.univ.r2 (48). The DNA primers used are listed in Table S3. The PCR products obtained were sequenced to analyze the nucleotide amplification specificity (ATGC Co., Ltd., Seoul, South Korea).

The whole-genome sequencing of *Bradyrhizobium* strains SPXBL-02, SPXBL-03, SPXBL-04, SMVTL-02, BLXBL-01, and BLXBL-03 was performed using genomic DNA extracted following the method described above. DNA quality was assessed to ensure that ≥50% of fragments were ≥30 kb and ≥90% were ≥10 kb, with concentrations measured using a Qubit fluorometer (Thermo Fisher Scientific). Genome sequencing was carried out using PacBio Revio HiFi technology, and assemblies were generated using the SMRT Link platform.

*Bradyrhizobium* sp. PMVTL-01 was cultivated in YM medium, and genomic DNA was extracted by Microbesng using SPRI beads (Beckman Coulter). DNA quality and genome sequencing were assessed following the method described above. The PMVTL-01 genome was sequenced using a hybrid strategy: Oxford Nanopore Technologies (ONT) sequencing, with DNA libraries prepared using Oxford Nanopore SQK-LSK109 kit and sequenced on a FLO-MIN106 (R.9.4.1) flow cell in a GridION (ONT, United Kingdom), followed by polishing with Illumina 250 bp paired-end reads generated using Nextera XT Library Prep Kit (Illumina, San Diego, USA). Hybrid assembly was performed using Unicycler version 0.4.0 (49). Genome assemblies were circularized using Circlator v1.5.5. Assembly metrics, including read N50, coverage, and contig circularity, are summarized in Table S4.

Putative T3Es were predicted using multiple complementary approaches: (i) identification of upstream *tts*-box promoter sequences using a Python-based motif search (https://github.com/tarnee-pp/bradyrhizobium_tts_finder); (ii) similarity-based screening with Effectidor (https://effectidor.tau.ac.il/index.html), which integrates multiple predictive features, including homology, machine learning classifiers and secretion signals; and (iii) BLASTP searches against a curated database of previously reported rhizobial Nop effectors. A curated database of *Bradyrhizobium* T3SS effectors (Nops) was assembled from experimentally validated proteins and well-annotated homologs in public databases (NCBI and MaGe). Sequence similarity searches (BLASTP, ≥50%) were used to identify candidate effectors in the analyzed genomes. Putative functions were inferred based on conserved domains using InterProScan, including SMART, PFAM, NCBIFAM, and Gene Ontology (GO) terms (24). The predicted T3Es and their features are listed in Table S5.

### Construction of a structural T3SS (*rhcN*) mutant

A single cross-homologous recombination technique was used to generate an insertion mutant in the *rhcN* gene (50), which is part of the T3SS in the *Bradyrhizobium* strains used in this study. An internal fragment of *rhcN* was amplified by PCR using the DNA primers listed in Table S2. The PCR product was digested with *XbaI* and *SalI* and then cloned into the corresponding sites of the plasmid pVO155-Sm/cefo-npt2-gfp (51). For biparental mating, the constructed plasmid was introduced into *E. coli* S17-1, which was then conjugated with *Bradyrhizobium* strains on AG or YM agar plates in a 1:1 ratio. Transconjugants were selected on AG or YM agar plates supplemented with 20 µg/mL cefo, 20 µg/mL Nal, 20 µg/mL Genta, and 100 µg/mL Km. The transconjugants were confirmed by PCR.

### Plant nodulation and symbiosis analysis

WT *Bradyrhizobium* and the Ω*rhcN* mutant strains were cultured in YM medium for five days. The cell cultures were then washed twice with 0.85% NaCl by centrifugation at 5,000 rpm for 10 min and adjusted to an optical density (OD) of 1.0 at 600 nm. Peanut (Kalasin2) seeds were surface sterilized with 95% ethanol for 20 s and 3% sodium hypochlorite for 20 s and then washed five times with sterile water. The seeds were soaked in distilled water for 4 h and germinated on sterile vermiculite for three days. Similarly, *Vigna* seeds (*V. radiata* CN72 and *V. mungo* CN80) were surface sterilized with 95% ethanol for 20 s and 3% sodium hypochlorite for 5 min and then washed five times with sterile water. The seeds were soaked in distilled water overnight and germinated on sterile water agar for two days (52). The seedlings were grown in Leonard’s jars containing sterilized vermiculite for five days before inoculation (45). Plants were watered with buffered nodulation medium (BNM) (53) and maintained in a growth chamber at 25°C under a 16 h light/8 h dark cycle for 30 days. The experiment was conducted in triplicate. Nodulation and nitrogen fixation were evaluated 30 days post-inoculation (dpi) by counting the number of nodules and performing an acetylene reduction assay (ARA) (45). Nodules were collected, incubated with 10% acetylene at 28°C for 1 h, and analyzed by gas chromatography. Ethylene and acetylene peak heights were measured using a PE-alumina packed column with an injector set at 150°C, an oven at 200°C, and a flame ionization detector (FID) at 50°C.

### Microscopy

Nodule phenotypes were examined using a stereomicroscope (Leica EZ4). Nodule development and the viability of bacteroids within symbiosomes were assessed with a Nikon Inverted Eclipse Ti-E Confocal Laser Scanning Microscope. For these analyses, nodules were embedded in 5% agarose and sectioned into 35–52  µm slices using a VT1000S vibratome (Leica, Nanterre, France). The sections were observed under a compound microscope (Carl Zeiss Primo Star HD) and subjected to staining protocols. For live/dead cell staining, the sections were incubated with SYTO9 (5  µM) and propidium iodide (PI, 30  µM) for 15 min. To detect cell wall components, a 1 × calcofluor white stain in 1 × phosphate-buffered saline buffer was applied for 5 min. Fluorescent signals were detected using specific emission filters: calcofluor (460–500  nm), SYTO9 (510–570  nm), and PI (600–650  nm).

### Statistical analyses

Statistical analyses were performed using one-way analysis of variance (ANOVA) with Tukey’s post hoc tests (*P*  ≤  0.05) in IBM SPSS Statistics 22.0. Independent *t*-tests were also conducted, and data visualization, including graph generation, was achieved using GraphPad Prism 8. The results are presented as mean values with standard deviations (SDs). The statistical significance is indicated as follows: ns = *P* > 0.05, *P ≤ 0.05, **P ≤ 0.01, ***P ≤ 0.001, and ****P ≤ 0.0001.

### Phylogenetic tree construction

The phylogenetic analyses of the 16S rRNA and *rhcN* genes were conducted by performing a BLAST search using the sequences of our strains against reference strains. The sequences showing the highest similarity to the reference strains were selected. In the case of *nodABC*, sequences were chosen from whole-genome annotations. Reference sequences were retrieved from the MicroScope platform (http://www.genoscope.cns.fr/agc/microscope) and the NCBI database (https://www.ncbi.nlm.nih.gov/protein/). Phylogenetic trees for the 16S rRNA, *rhcN* gene, and *nodABC* cluster were constructed using MEGA (Molecular Evolutionary Genetics Analysis) software version 11.0. The trees were generated using the maximum likelihood method under the Kimura 2-parameter (K2P) model with a discrete Gamma distribution (+G) and five rate categories. Statistical support for the tree nodes was assessed using bootstrap analysis with 1,000 replicates. A phylogenomic tree was constructed using IQ-tree v2.2.0.3 (54) based on a multi-sequence alignment of 718 genes of the core genome shared by at least 98% of *Bradyrhizobium* strains, generated by PPanGGolin v1.2.63 (55). The phylogeny was inferred from the resulting alignment under the model recommended by ModelFinder (56), and branch supports with ultrafast bootstrap were estimated from 100,000 iterations (57). Final editing was carried out using iTOL (https://itol.embl.de/) (58). ANI analysis, including alignment coverage, alignment lengths, and Hadamard transformation, was performed using the ANIm mode of the PyANI Python module, which is based on MUMmer (https://github.com/widdowquinn/pyani) (59).

## Data Availability

The GenBank accession numbers for the 16S rRNA gene sequences of strains SPXBL-05, SPXBL-06, PMVTL-02, and BLXBL-02 are PV012544, PV012545, PV012547, and PV012546, respectively. The accession numbers for the *rhcN* gene sequences of strains SPXBL-05, SPXBL-06, PMVTL-02, and BLXBL-02 are PV132762, PV132763, PV132765, and PV132764, respectively. The whole-genome projects for strains PMVTL-01, SPXBL-02, SPXBL-03, SPXBL-04, SMVTL-02, BLXBL-01, and BLXBL-03 were deposited in the NCBI Sequence Read Archive under BioProject accession number PRJNA1215578, with accession numbers CP193982, CP182474, CP182473, CP182472, CP182469, CP182471, and CP182470, respectively. The whole-genome sequences were also deposited in the MicroScope platform (http://www.genoscope.cns.fr/agc/microscope). The code and scripts used for genome assembly, annotation, and generation are presented in Table S4. The data sets generated and/or analyzed during the current study are available from the corresponding author upon reasonable request.

## SUPPLEMENTAL MATERIAL

Supplemental material aem.00600-25-s0001.docx Fig. S1 to S5 and Tables S1 to S3.

Table S4 aem.00600-25-s0002.xlsx Summary of PacBio HiFi reads and assembly statistics for *Bradyrhizobium* strains.

Table S5 aem.00600-25-s0003.xlsx Summary of predicted Type III effectors (T3Es) in *Bradyrhizobium* sp.

ASM does not own the copyrights to Supplemental Material that may be linked to, or accessed through, an article. The authors have granted ASM a non-exclusive, world-wide license to publish the Supplemental Material files. Please contact the corresponding author directly for reuse.

## References

[1] Clúa J, Roda C, Zanetti ME, Blanco FA. 2018. Compatibility between legumes and rhizobia for the establishment of a successful nitrogen-fixing symbiosis. Genes (Basel) 9:125. doi:10.3390/genes9030125 29495432 PMC5867846

[2] Oldroyd GED. 2013. Speak, friend, and enter: signalling systems that promote beneficial symbiotic associations in plants. Nat Rev Microbiol 11:252–263. doi:10.1038/nrmicro2990 23493145

[3] Krause A, Doerfel A, Göttfert M. 2002. Mutational and transcriptional analysis of the type III secretion system of Bradyrhizobium japonicum. Mol Plant Microbe Interact 15:1228–1235. doi:10.1094/MPMI.2002.15.12.1228 12481995

[4] Ausmees N, Kobayashi H, Deakin WJ, Marie C, Krishnan HB, Broughton WJ, Perret X. 2004. Characterization of NopP, a type III secreted effector of Rhizobium sp. strain NGR234. J Bacteriol 186:4774–4780. doi:10.1128/JB.186.14.4774-4780.2004 15231809 PMC438593

[5] Wassem R, Kobayashi H, Kambara K, Le Quéré A, Walker GC, Broughton WJ, Deakin WJ. 2008. TtsI regulates symbiotic genes in Rhizobium species NGR234 by binding to tts boxes. Mol Microbiol 68:736–748. doi:10.1111/j.1365-2958.2008.06187.x 18363648 PMC2770584

[6] Piromyou P, Nguyen HP, Songwattana P, Boonchuen P, Teamtisong K, Tittabutr P, Boonkerd N, Alisha Tantasawat P, Göttfert M, Okazaki S, Teaumroong N. 2021. The Bradyrhizobium diazoefficiens type III effector NopE modulates the regulation of plant hormones towards nodulation in Vigna radiata. Sci Rep 11:16604. doi:10.1038/s41598-021-95925-4 34400661 PMC8367979

[7] Dai W-J, Zeng Y, Xie Z-P, Staehelin C. 2008. Symbiosis-promoting and deleterious effects of NopT, a novel type 3 effector of Rhizobium sp. strain NGR234. J Bacteriol 190:5101–5110. doi:10.1128/JB.00306-08 18487326 PMC2447009

[8] Skorpil P, Saad MM, Boukli NM, Kobayashi H, Ares-Orpel F, Broughton WJ, Deakin WJ. 2005. NopP, a phosphorylated effector of Rhizobium sp. strain NGR234, is a major determinant of nodulation of the tropical legumes Flemingia congesta and Tephrosia vogelii*.* Mol Microbiol 57:1304–1317. doi:10.1111/j.1365-2958.2005.04768.x 16102002

[9] Jiménez-Guerrero I, Pérez-Montaño F, Medina C, Ollero FJ, López-Baena FJ. 2017. The Sinorhizobium (Ensifer) fredii HH103 nodulation outer protein NopI is a determinant for efficient nodulation of soybean and cowpea plants. Appl Environ Microbiol 83:e02770-16. doi:10.1128/AEM.02770-16 27986730 PMC5311403

[10] Piromyou P, Pruksametanan N, Nguyen HP, Songwattana P, Wongdee J, Nareephot P, Greetatorn T, Teamtisong K, Tittabutr P, Boonkerd N, Sato S, Boonchuen P, Okazaki S, Teaumroong N. 2024. NopP2 effector of Bradyrhizobium elkanii USDA61 is a determinant of nodulation in Vigna radiata cultivars. Sci Rep 14:24541. doi:10.1038/s41598-024-75294-4 39424841 PMC11489812

[11] Songwattana P, Chaintreuil C, Wongdee J, Teulet A, Mbaye M, Piromyou P, Gully D, Fardoux J, Zoumman AMA, Camuel A, Tittabutr P, Teaumroong N, Giraud E. 2021. Identification of type III effectors modulating the symbiotic properties of Bradyrhizobium vignae strain ORS3257 with various Vigna species. Sci Rep 11:4874. doi:10.1038/s41598-021-84205-w 33649428 PMC7921652

[12] Sugawara M, Epstein B, Badgley BD, Unno T, Xu L, Reese J, Gyaneshwar P, Denny R, Mudge J, Bharti AK, Farmer AD, May GD, Woodward JE, Médigue C, Vallenet D, Lajus A, Rouy Z, Martinez-Vaz B, Tiffin P, Young ND, Sadowsky MJ. 2013. Comparative genomics of the core and accessory genomes of 48 Sinorhizobiumstrains comprising five genospecies. Genome Biol 14:2–R17. doi:10.1186/gb-2013-14-2-r17 PMC4053727 23425606

[13] Aphaiso B, Piromyou P, Boonchuen P, Songwattana P, Wongdee J, Greetatorn T, Teamtisong K, Camuel A, Tittabutr P, Boonkerd N, Giraud E, Teaumroong N. 2024. A new type III effector from Bradyrhizobium sp. DOA9 encoding a putative SUMO-protease blocks nodulation in Arachis hypogaea L. Sci Rep 14:31646. doi:10.1038/s41598-024-78913-2 39738104 PMC11685577

[14] Jaiswal SK, Msimbira LA, Dakora FD. 2017. Phylogenetically diverse group of native bacterial symbionts isolated from root nodules of groundnut (Arachis hypogaea L.) in South Africa. Syst Appl Microbiol 40:215–226. doi:10.1016/j.syapm.2017.02.002 28372899 PMC5460907

[15] Chang YL, Wang JY, Wang ET, Liu HC, Sui XH, Chen WX. 2011. Bradyrhizobium lablabi sp. nov., isolated from effective nodules of Lablab purpureus and Arachis hypogaea. Int J Syst Evol Microbiol 61:2496–2502. doi:10.1099/ijs.0.027110-0 21112989

[16] Wang R, Chang YL, Zheng WT, Zhang D, Zhang XX, Sui XH, Wang ET, Hu JQ, Zhang LY, Chen WX. 2013. Bradyrhizobium arachidis sp. nov., isolated from effective nodules of Arachis hypogaea grown in China. Syst Appl Microbiol 36:101–105. doi:10.1016/j.syapm.2012.10.009 23295123

[17] Phimphong T. 2022. Selection of Bradyrhizobia for peanut production in the LAO People’s Democratic Republic. MSc Thesis, School of Biotechnology Institute of Agricultural Technology Suranaree

[18] Phimphong T, Sibounnavong P, Phommalath S, Wongdee J, Songwattana P, Piromyou P, Greetatorn T, Boonkerd N, Tittabutr P, Teaumroong N. 2023. Selection and evaluation of Bradyrhizobium inoculum for peanut, Arachis hypogea production in the Lao People’s Democratic Republic. JANS 15:137–154. doi:10.31018/jans.v15i1.4270

[19] Teulet A, Gully D, Rouy Z, Camuel A, Koebnik R, Giraud E, Lassalle F. 2020. Phylogenetic distribution and evolutionary dynamics of nod and T3SS genes in the genus Bradyrhizobium. Microb Genom 6:e000407. doi:10.1099/mgen.0.000407 PMC7643967 32783800

[20] Camuel A, Teulet A, Carcagno M, Haq F, Pacquit V, Gully D, Pervent M, Chaintreuil C, Fardoux J, Horta-Araujo N, Okazaki S, Ratu STN, Gueye F, Zilli J, Nouwen N, Arrighi J-F, Luo H, Mergaert P, Deslandes L, Giraud E. 2023. Widespread Bradyrhizobium distribution of diverse type III effectors that trigger legume nodulation in the absence of Nod factor. ISME J 17:1416–1429. doi:10.1038/s41396-023-01458-1 37355742 PMC10432411

[21] Okazaki S, Okabe S, Higashi M, Shimoda Y, Sato S, Tabata S, Hashiguchi M, Akashi R, Göttfert M, Saeki K. 2010. Identification and functional analysis of type III effector proteins in Mesorhizobium loti. Mol Plant Microbe Interact 23:223–234. doi:10.1094/MPMI-23-2-0223 20064065

[22] Xiang Q-W, Bai J, Cai J, Huang Q-Y, Wang Y, Wagner C, Zhong Z, Liang Y. 2015. NopD of Bradyrhizobium sp. XS1150 possesses SUMO protease activity. Front Microbiol 11 10.3389/fmicb.2020.00386 PMC7098955 32265858

[23] Staehelin C, Krishnan HB. 2015. Nodulation outer proteins: double-edged swords of symbiotic rhizobia. Biochem J 470:263–274. doi:10.1042/BJ20150518 26341483

[24] Jones P, Binns D, Chang H-Y, Fraser M, Li W, McAnulla C, McWilliam H, Maslen J, Mitchell A, Nuka G, Pesseat S, Quinn AF, Sangrador-Vegas A, Scheremetjew M, Yong S-Y, Lopez R, Hunter S. 2014. InterProScan 5: genome-scale protein function classification. Bioinformatics 30:1236–1240. doi:10.1093/bioinformatics/btu031 24451626 PMC3998142

[25] Nguyen HP, Ratu STN, Yasuda M, Göttfert M, Okazaki S. 2018. InnB, a novel type IIi effector of Bradyrhizobium elkanii USDA61, controls symbiosis with Vigna species. Front Microbiol 9:3155. doi:10.3389/fmicb.2018.03155 30619219 PMC6305347

[26] Kaneko T, Nakamura Y, Sato S, Asamizu E, Kato T, Sasamoto S, Watanabe A, Idesawa K, Ishikawa A, Kawashima K, Kimura T, Kishida Y, Kiyokawa C, Kohara M, Matsumoto M, Matsuno A, Mochizuki Y, Nakayama S, Nakazaki N, Shimpo S, Sugimoto M, Takeuchi C, Yamada M, Tabata S. 2000. Complete genome structure of the nitrogen-fixing symbiotic bacterium Mesorhizobium loti. DNA Res 7:331–338. doi:10.1093/dnares/7.6.331 11214968

[27] Nguyen HP, Ratu STN, Yasuda M, Teaumroong N, Okazaki S. 2020. Identification of Bradyrhizobium elkanii USDA61 type III effectors determining symbiosis with Vigna mungo. Genes (Basel) 11:474. doi:10.3390/genes11050474 32349348 PMC7291247

[28] Patra D, Pal KK, Mandal S. 2024. Inter-species interaction of Bradyrhizobia affects their colonization and plant growth promotion in Arachis hypogaea. World J Microbiol Biotechnol 40:234. doi:10.1007/s11274-024-04035-6 38844667

[29] Wu Y, Li YH, Shang JY, Wang ET, Chen L, Huo B, Sui XH, Tian CF, Chen WF, Chen WX. 2020. Multiple genes of symbiotic plasmid and chromosome in type II peanut Bradyrhizobium strains corresponding to the incompatible symbiosis with Vigna radiata. Front Microbiol 11. doi:10.3389/fmicb.2020.01175 PMC7324677 32655513

[30] Kaneko T, Maita H, Hirakawa H, Uchiike N, Minamisawa K, Watanabe A, Sato S. 2011. Complete genome sequence of the soybean symbiont Bradyrhizobium japonicum strain USDA6T. Genes (Basel) 2:763–787. doi:10.3390/genes2040763 24710291 PMC3927601

[31] Zhong C, Hu G, Hu C, Xu C, Zhang Z, Ning K. 2024. Comparative genomics analysis reveals genetic characteristics and nitrogen fixation profile of Bradyrhizobium. iScience 27:108948. doi:10.1016/j.isci.2024.108948 38322985 PMC10845061

[32] Wasai-Hara S, Minamisawa K, Cloutier S, Bromfield ESP. 2020. Strains of Bradyrhizobium cosmicum sp. nov., isolated from contrasting habitats in Japan and Canada possess photosynthesis gene clusters with the hallmark of genomic islands. Int J Syst Evol Microbiol 70:5063–5074. doi:10.1099/ijsem.0.004380 32804606 PMC7656271

[33] Noisangiam R, Teamtisong K, Tittabutr P, Boonkerd N, Toshiki U, Minamisawa K, Teaumroong N. 2012. Genetic diversity, symbiotic evolution, and proposed infection process of Bradyrhizobium strains isolated from root nodules of Aeschynomene americana L. in Thailand. Appl Environ Microbiol 78:6236–6250. doi:10.1128/AEM.00897-12 22752179 PMC3416612

[34] Piromyou P, Songwattana P, Teamtisong K, Tittabutr P, Boonkerd N, Tantasawat PA, Giraud E, Göttfert M, Teaumroong N. 2019. Mutualistic co-evolution of T3SSs during the establishment of symbiotic relationships between Vigna radiata and Bradyrhizobia. Microbiologyopen 8:e00781. doi:10.1002/mbo3.781 30628192 PMC6612562

[35] Silva FV, De Meyer SE, Simões-Araújo JL, Barbé T da C, Xavier GR, O’Hara G, Ardley JK, Rumjanek NG, Willems A, Zilli JE. 2014. Bradyrhizobium manausense sp. nov., isolated from effective nodules of Vigna unguiculata grown in Brazilian Amazonian rainforest soils. Int J Syst Evol Microbiol 64:2358–2363. doi:10.1099/ijs.0.061259-0 24744018

[36] Lohou D, Turner M, Lonjon F, Cazalé A-C, Peeters N, Genin S, Vailleau F. 2014. HpaP modulates type III effector secretion in Ralstonia solanacearum and harbours a substrate specificity switch domain essential for virulence. Mol Plant Pathol 15:601–614. doi:10.1111/mpp.12119 24405562 PMC6638691

[37] Liu P, Zhang W, Zhang L-Q, Liu X, Wei H-L. 2016. Supramolecular structure and functional analysis of the type III secretion system in pseudomonas fluorescens 2P24. Front Plant Sci 6. doi:10.3389/fpls.2015.01190 PMC4700148 26779224

[38] Figueira R, Watson KG, Holden DW, Helaine S. 2013. Identification of salmonella pathogenicity island-2 type III secretion system effectors involved in intramacrophage replication of S. enterica serovar typhimurium: implications for rational vaccine design. MBio 4:e00065. doi:10.1128/mBio.00065-13 23592259 PMC3634603

[39] Hotson A, Chosed R, Shu H, Orth K, Mudgett MB. 2003. Xanthomonas type III effector XopD targets SUMO-conjugated proteins in planta. Mol Microbiol 50:377–389. doi:10.1046/j.1365-2958.2003.03730.x 14617166

[40] Rodrigues JA, López-Baena FJ, Ollero FJ, Vinardell JM, Espuny M del R, Bellogín RA, Ruiz-Sainz JE, Thomas JR, Sumpton D, Ault J, Thomas-Oates J. 2007. NopM and NopD are rhizobial nodulation outer proteins: identification using LC-MALDI and LC-ESI with a monolithic capillary column. J Proteome Res 6:1029–1037. doi:10.1021/pr060519f 17249710

[41] Tsurumaru H, Hashimoto S, Okizaki K, Kanesaki Y, Yoshikawa H, Yamakawa T. 2015. A putative type III secretion system effector encoded by the MA20_12780 gene in Bradyrhizobium japonicum Is-34 causes incompatibility with Rj4 genotype soybeans. Appl Environ Microbiol 81:5812–5819. doi:10.1128/AEM.00823-15 26092458 PMC4551253

[42] Sugawara M, Takahashi S, Umehara Y, Iwano H, Tsurumaru H, Odake H, Suzuki Y, Kondo H, Konno Y, Yamakawa T, Sato S, Mitsui H, Minamisawa K. 2018. Variation in bradyrhizobial NopP effector determines symbiotic incompatibility with Rj2-soybeans via effector-triggered immunity. Nat Commun 9:1. doi:10.1038/s41467-018-05663-x 30087346 PMC6081438

[43] Okazaki S, Zehner S, Hempel J, Lang K, Göttfert M. 2009. Genetic organization and functional analysis of the type III secretion system of Bradyrhizobium elkanii. FEMS Microbiol Lett 295:88–95. doi:10.1111/j.1574-6968.2009.01593.x 19473255

[44] Sadowsky MJ, Tully RE, Cregan PB, Keyser HH. 1987. Genetic diversity in Bradyrhizobium japonicum serogroup 123 and its relation to genotype-specific nodulation of soybean. Appl Environ Microbiol 53:2624–2630. doi:10.1128/aem.53.11.2624-2630.1987 16347481 PMC204163

[45] Somasegaran P, Hoben HJ. 1994. Handbook for rhizobia: methods in legume-rhizobium technology

[46] Wood EJ. 1983. Molecular cloning. a laboratory manual. Edited by E. F. Fritsch Maniatis and J. Sambrook. Biochem Educ 11:82. doi:10.1016/0307-4412(83)90068-7

[47] Weisburg WG, Barns SM, Pelletier DA, Lane DJ. 1991. 16S ribosomal DNA amplification for phylogenetic study. J Bacteriol 173:697–703. doi:10.1128/jb.173.2.697-703.1991 1987160 PMC207061

[48] Okazaki S, Tittabutr P, Teulet A, Thouin J, Fardoux J, Chaintreuil C, Gully D, Arrighi J-F, Furuta N, Miwa H, Yasuda M, Nouwen N, Teaumroong N, Giraud E. 2016. Rhizobium-legume symbiosis in the absence of Nod factors: two possible scenarios with or without the T3SS. ISME J 10:64–74. doi:10.1038/ismej.2015.103 26161635 PMC4681849

[49] Wick RR, Judd LM, Gorrie CL, Holt KE. 2017. Unicycler: resolving bacterial genome assemblies from short and long sequencing reads. PLOS Comput Biol 13:e1005595. doi:10.1371/journal.pcbi.1005595 28594827 PMC5481147

[50] Songwattana P, Noisangiam R, Teamtisong K, Prakamhang J, Teulet A, Tittabutr P, Piromyou P, Boonkerd N, Giraud E, Teaumroong N. 2017. Type 3 secretion system (T3SS) of Bradyrhizobium sp. DOA9 and its roles in legume symbiosis and rice endophytic association. Front Microbiol 8:1810. doi:10.3389/fmicb.2017.01810 28979252 PMC5611442

[51] Wongdee J, Songwattana P, Nouwen N, Noisangiam R, Fardoux J, Chaintreuil C, Teaumroong N, Tittabutr P, Giraud E. 2016. nifDK clusters located on the chromosome and megaplasmid of Bradyrhizobium sp. strain DOA9 contribute differently to nitrogenase activity during symbiosis and free-living growth. Mol Plant Microbe Interact 29:767–773. doi:10.1094/MPMI-07-16-0140-R 27603559

[52] Teamtisong K, Songwattana P, Noisangiam R, Piromyou P, Boonkerd N, Tittabutr P, Minamisawa K, Nantagij A, Okazaki S, Abe M, Uchiumi T, Teaumroong N. 2014. Divergent nod-containing Bradyrhizobium sp. DOA9 with a megaplasmid and its host range. Microbes Environ 29:370–376. doi:10.1264/jsme2.ME14065 25283477 PMC4262360

[53] Ehrhardt DW, Atkinson EM, Long SR. 1992. Depolarization of alfalfa root hair membrane potential by Rhizobium meliloti Nod factors. Science 256:998–1000. doi:10.1126/science.10744524 10744524

[54] Nguyen L-T, Schmidt HA, von Haeseler A, Minh BQ. 2015. IQ-TREE: a fast and effective stochastic algorithm for estimating maximum-likelihood phylogenies. Mol Biol Evol 32:268–274. doi:10.1093/molbev/msu300 25371430 PMC4271533

[55] Gautreau G, Bazin A, Gachet M, Planel R, Burlot L, Dubois M, Perrin A, Médigue C, Calteau A, Cruveiller S, Matias C, Ambroise C, Rocha EPC, Vallenet D. 2020. PPanGGOLiN: Depicting microbial diversity via a partitioned pangenome graph. PLOS Comput Biol 16:e1007732. doi:10.1371/journal.pcbi.1007732 32191703 PMC7108747

[56] Kalyaanamoorthy S, Minh BQ, Wong TKF, von Haeseler A, Jermiin LS. 2017. ModelFinder: fast model selection for accurate phylogenetic estimates. Nat Methods 14:587–589. doi:10.1038/nmeth.4285 28481363 PMC5453245

[57] Hoang DT, Chernomor O, von Haeseler A, Minh BQ, Vinh LS. 2018. UFBoot2: improving the ultrafast bootstrap approximation. Mol Biol Evol 35:518–522. doi:10.1093/molbev/msx281 29077904 PMC5850222

[58] Letunic I, Bork P. 2021. Interactive Tree Of Life (iTOL) v5: an online tool for phylogenetic tree display and annotation. Nucleic Acids Res 49:W293–W296. doi:10.1093/nar/gkab301 33885785 PMC8265157

[59] Pritchard L, Glover RH, Humphris S, Elphinstone JG, Toth IK. 2016. Genomics and taxonomy in diagnostics for food security: soft-rotting enterobacterial plant pathogens. Anal Methods 8:12–24. doi:10.1039/C5AY02550H

