# Supplementary material for: Diversity of bradyrhizobial T3SS systems and their roles in symbiosis with peanut (Arachis hypogaea) and Vigna species (V. radiata and V. mungo)
Source: Appl Environ Microbiol. 2025 Aug 8;91(9):e00600-25. doi: 10.1128/aem.00600-25 (PMC12442349; doi:10.1128/aem.00600-25)
Supplement: Supplemental material — Fig. S1 to S5 and Tables S1 to S3. [file aem.00600-25-s0001.docx]

**Supplementary Material**

**Diversity of bradyrhizobial T3SS systems and their roles in symbiosis with peanut (*Arachis hypogaea*) and *Vigna* species (*V. radiata* and *V. mungo*)**

Tarnee Phimphong ^1,2^ and Shun Hashimoto ^3^, Pongpan Songwattana ^4^, Jenjira Wongdee ^4^, Teerana Greetatorn ^4^, Kamonluck Teamtisong ^5^, Pakpoom Boonchuen ^1^, Sachiko Masuda ^6^, Arisa Shibata ^6^, Ken Shirasu ^6^, Phoutthasone Sibounnavong ^2^, Panlada Tittabutr ^1^, Nantakorn Boonkerd ^1^, Shusei Sato ^3^, Djamel Gully ^7^, Eric Graud ^7^, Pongdet Piromyou ^4*^, Neung Teaumroong ^1*^

^1^School of Biotechnology, Institute of Agricultural Technology, Suranaree University of Technology, Nakhon Ratchasima 30000, Thailand

^2^Faculty of Agriculture, National University of Laos, P. O. Box 7322, Vientiane capital, Lao PDR

^3^Graduate School of Life Sciences, Tohoku University Symbiosis Genomics Lab. 2-1-1 Katahira, Aoba, Sendai 980-8577, Japan

^4^Institute of Research and Development, Suranaree University of Technology, Nakhon Ratchasima 30000, Thailand

^5^The Center for Scientific and Technological Equipment, Suranaree University of ^Technology, Nakhon Ratchasima 30000, Thailand^

^6^RIKEN Center for Sustainable Resource Science, RIKEN-TRIP, 1-7-22 Suehiro-cho, Tsurumi, Yokohama, Kanagawa, 230-0045, Japan

^7^IRD, Laboratoire des Symbioses Tropicales et Méditerranéennes, UMR 113, IRD/CIRAD/INRAE/Université de Montpellier/SupAgro, Campus de Baillarguet, TA-A82/J, 34398, Montpellier Cedex 5, France


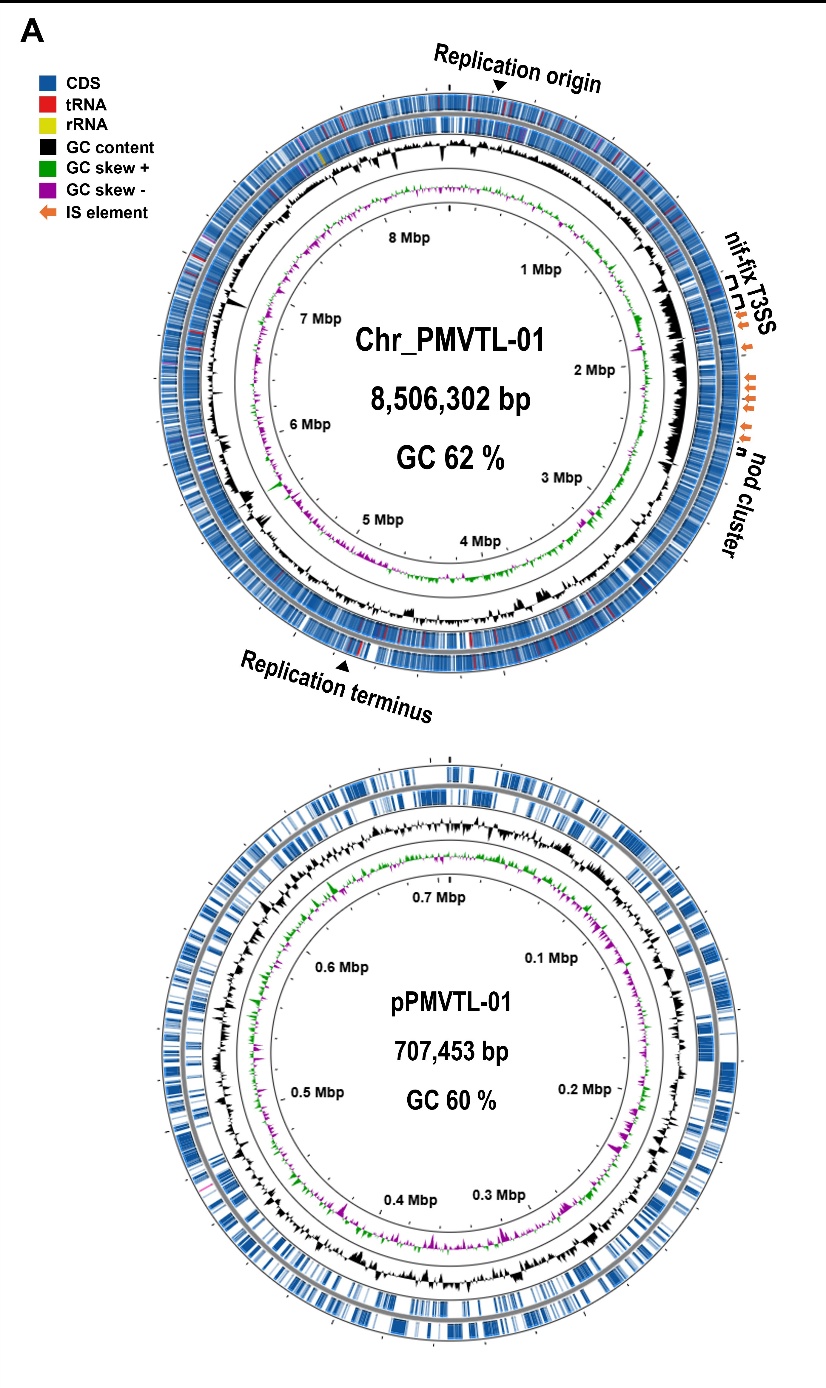


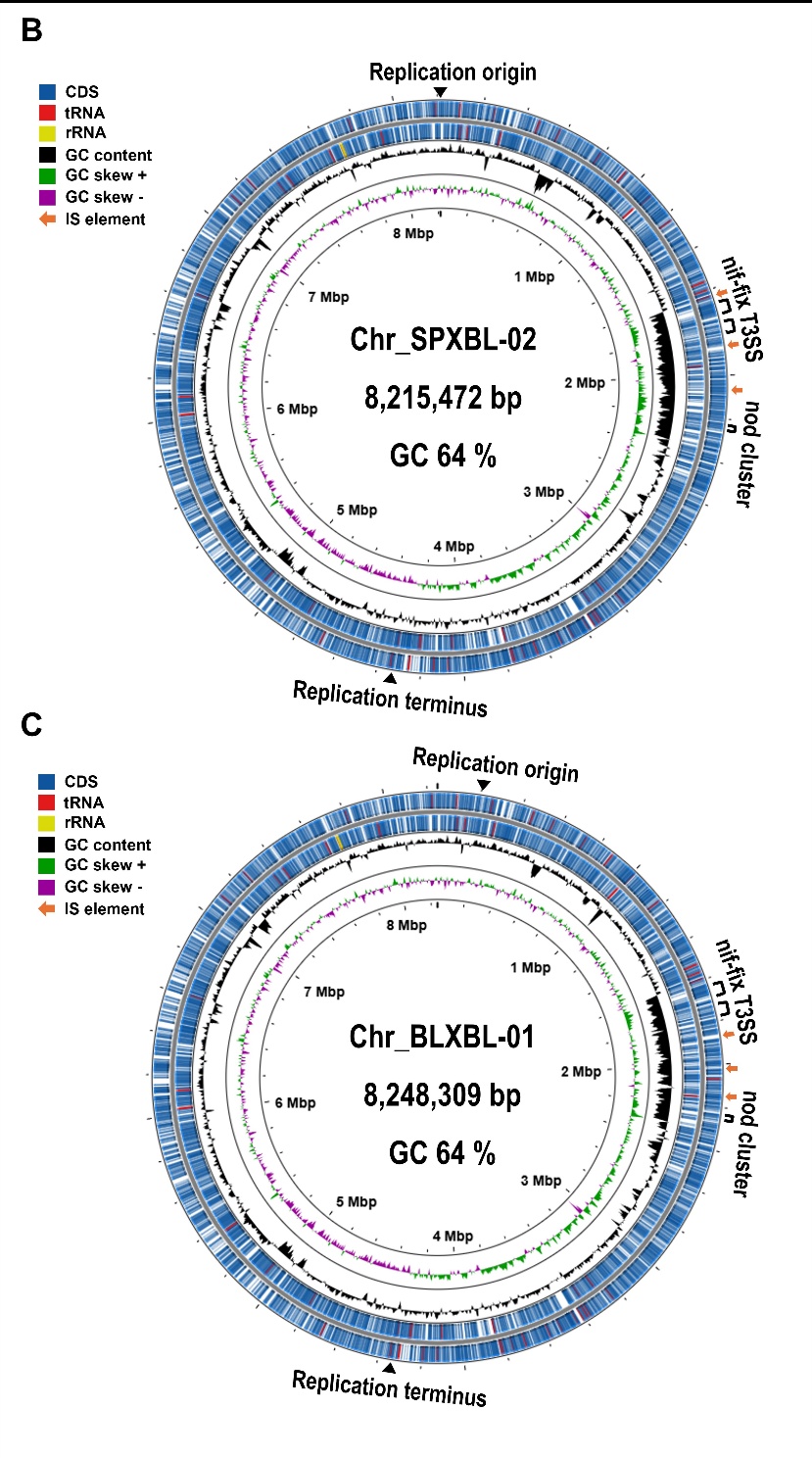


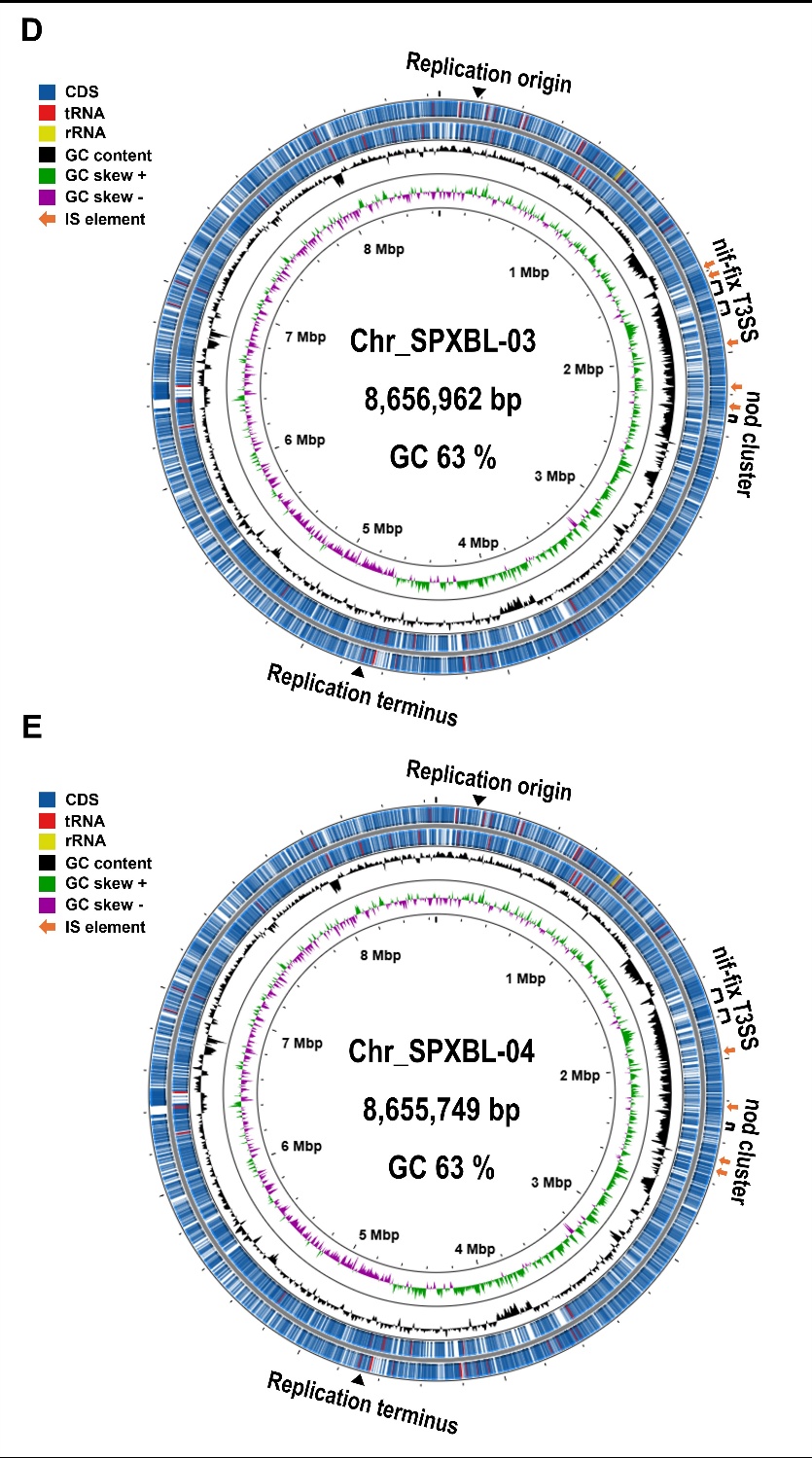


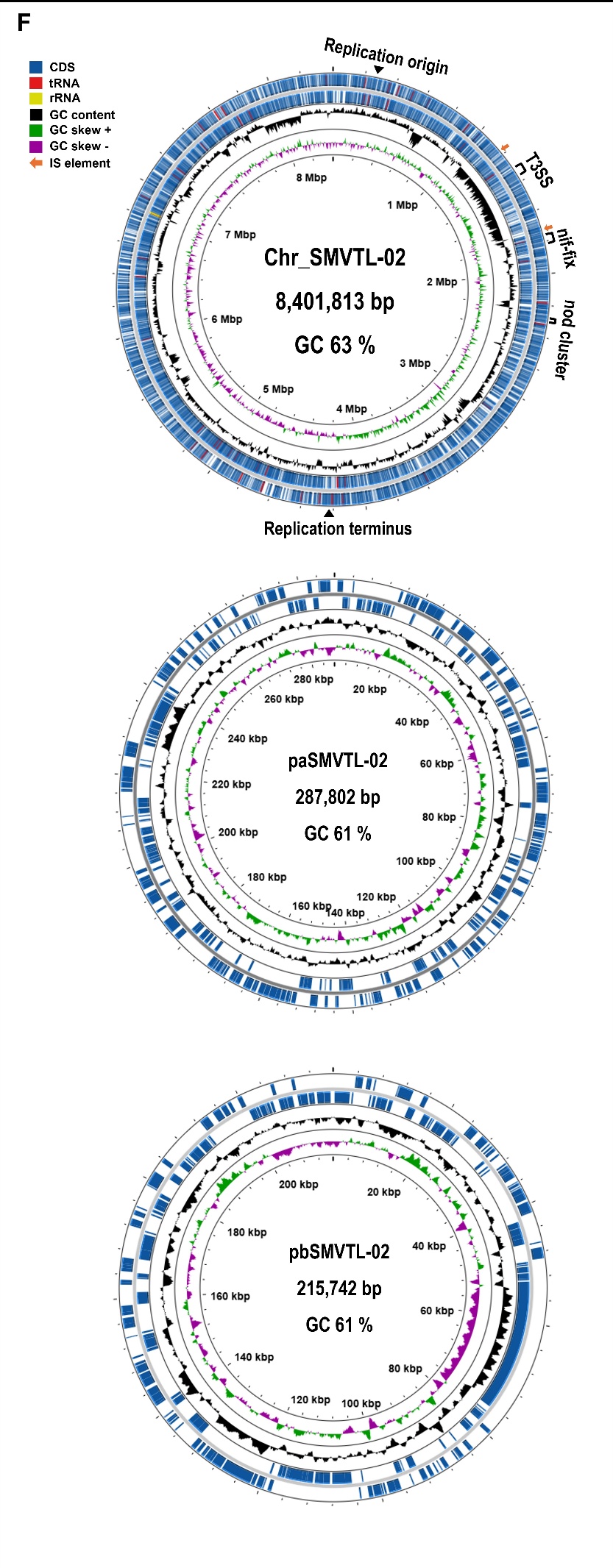


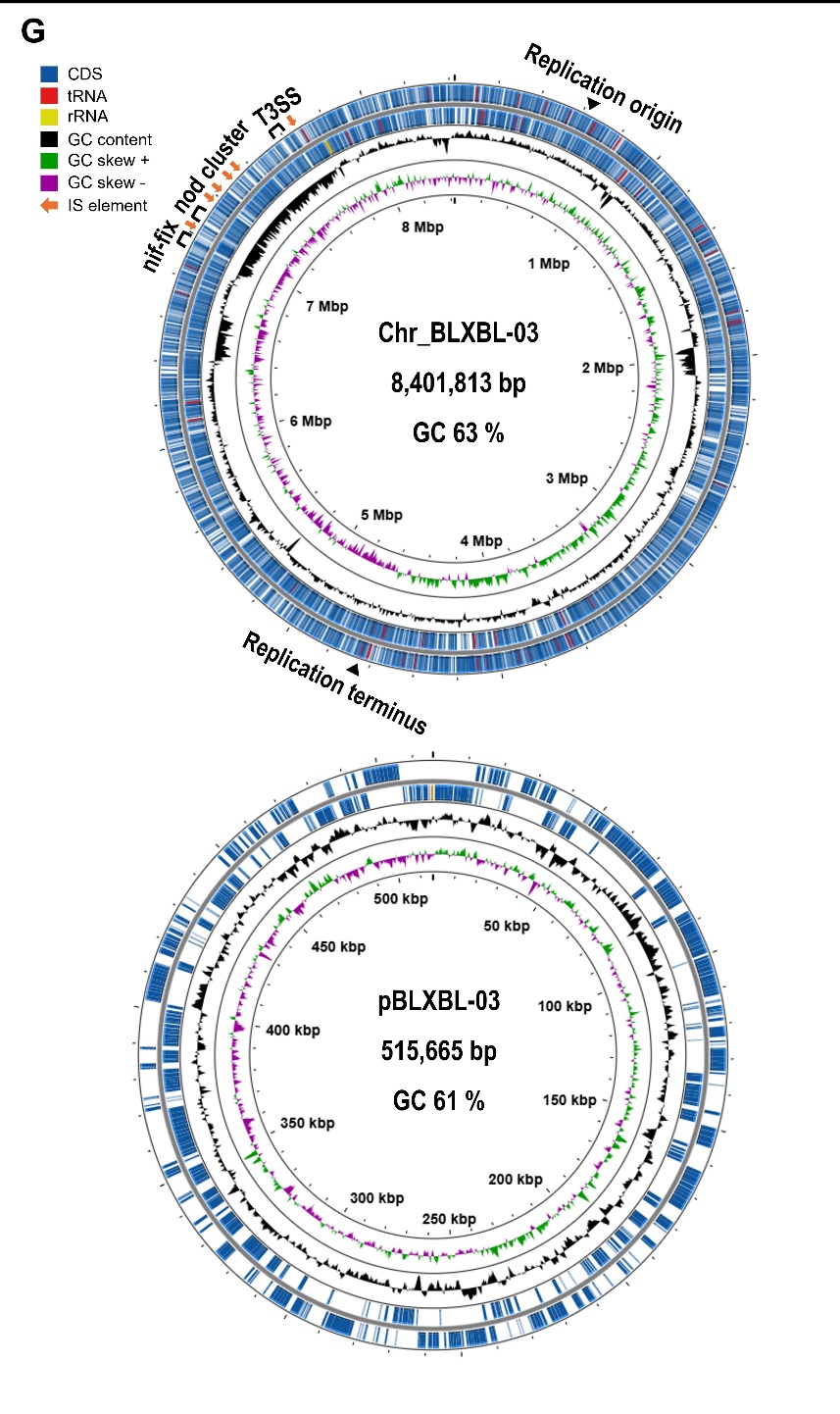


**Fig. S1** Genome structure of seven *Bradyrhizobium* sp. strains.The circular chromosome and plasmid structures were generated using Proksee - Genome Analysis. **(A)** G1: PMVTL-01. **(B, C)** G2: SPXBL-02 and BLXBL-01. **(D, E)** G3: SPXBL-03 and SPXBL-04. **(F)** G4: SMVTL-02. **(G)** G5: BLXBL-03. The GC content ring shows how the GC content varies from the average (higher GC content is green, lower is purple). The innermost ring marks genome positions in megabases (Mb). The outermost ring highlights the locations of T3SS, *nod* genes, *nif-fix* gene clusters, replication origin and replication terminus.


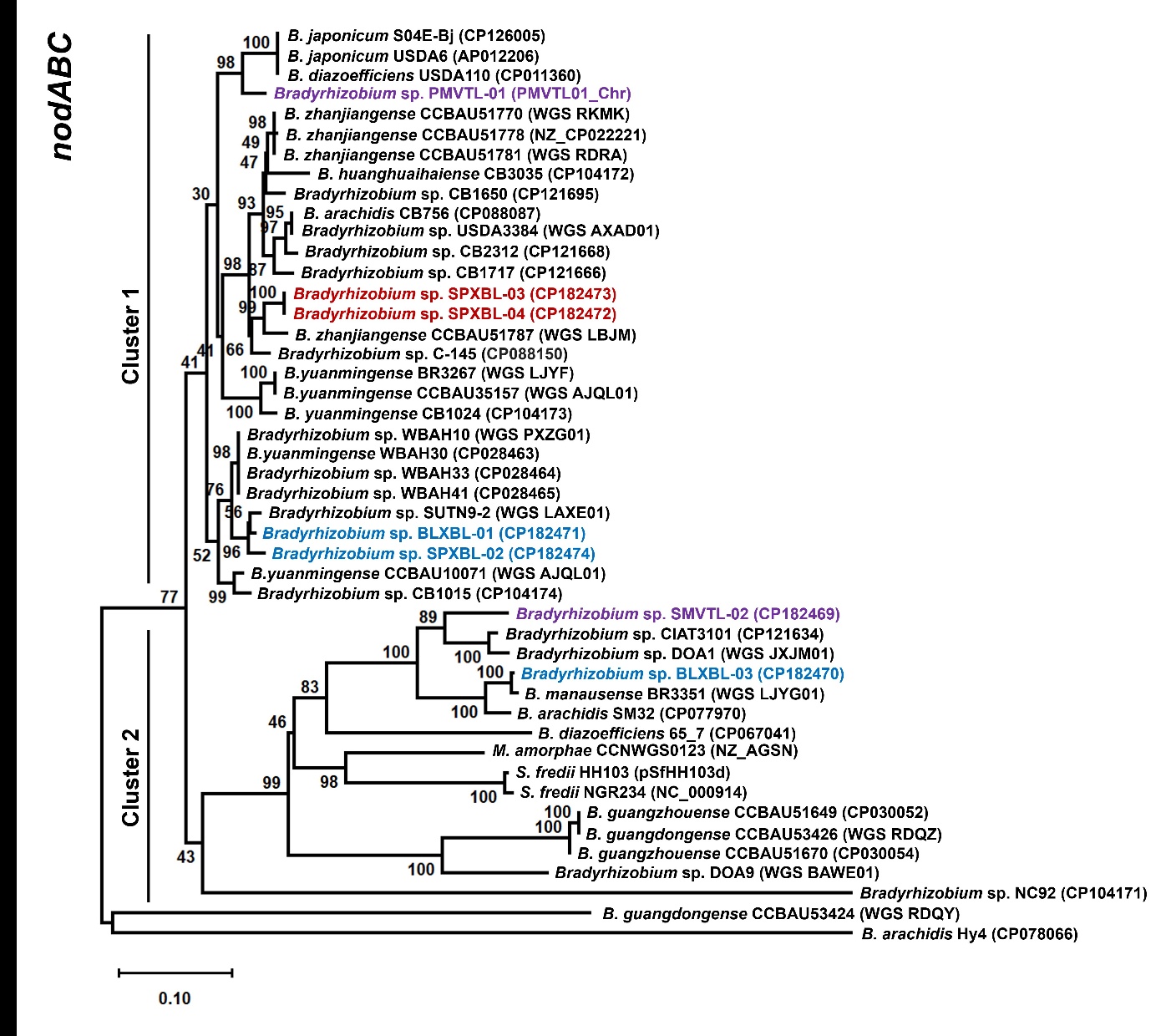


**Fig. S2** Phylogenetic tree of the *nodABC* gene Cluster of 46 rhizobial strains. The phylogenetic tree shows the evolutionary relationships of the *nodABC* gene cluster among *Br*adyrhizobium and related species (Color: blue 16s-clade 1, purple 16s-clade 2 and red 16s-clade 3). Tree was constructed using the maximum likelihood (ML) method, following the Kimura 2-parameter (K2P) model with a Gamma distribution (+G) and five rate categories. Bootstrap analysis with 1,000 replicates was used to assess statistical support for the tree nodes. The scale bar represents 0.10% sequence divergence, reflecting the genetic differences among the analyzed strains.


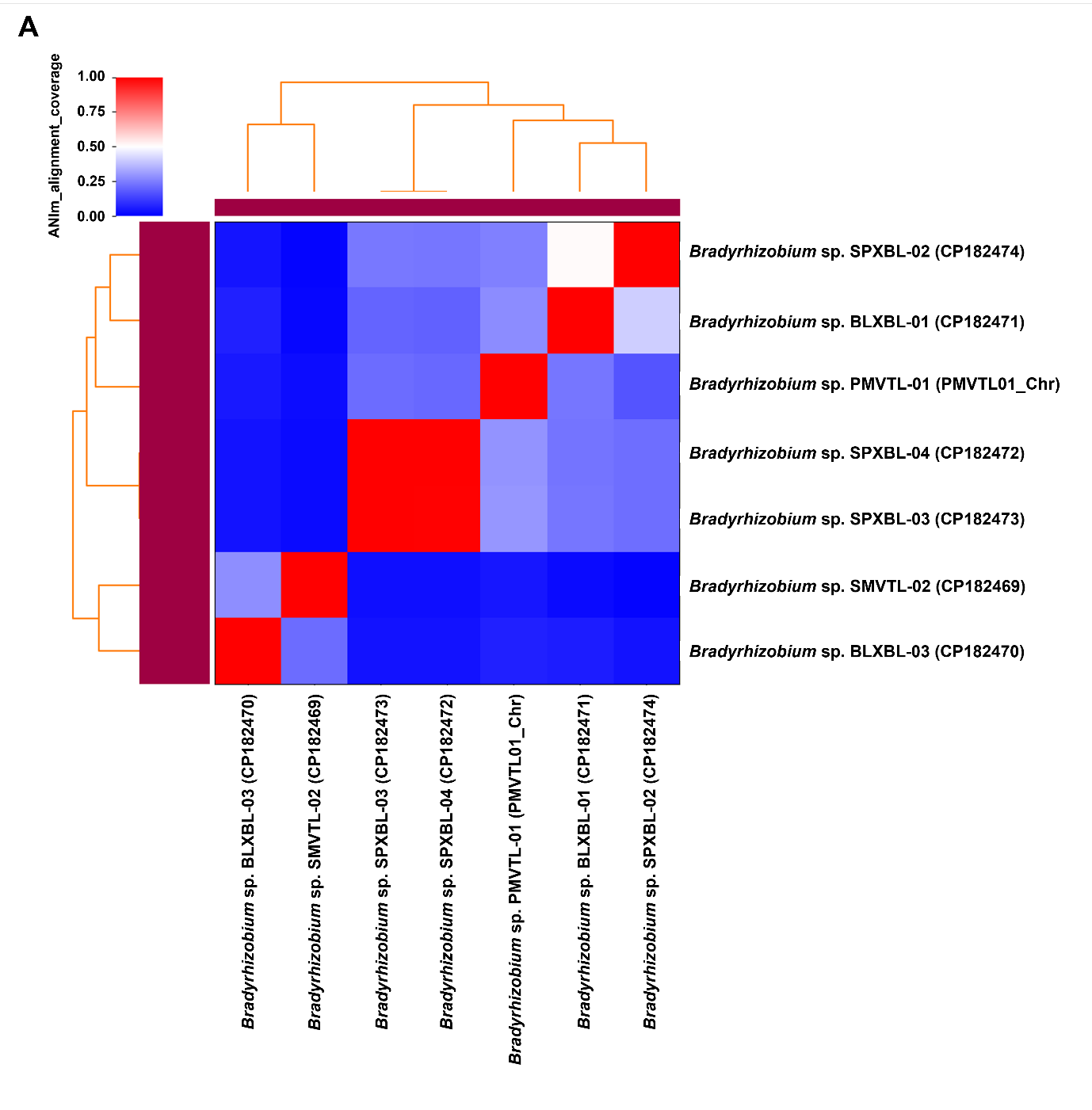


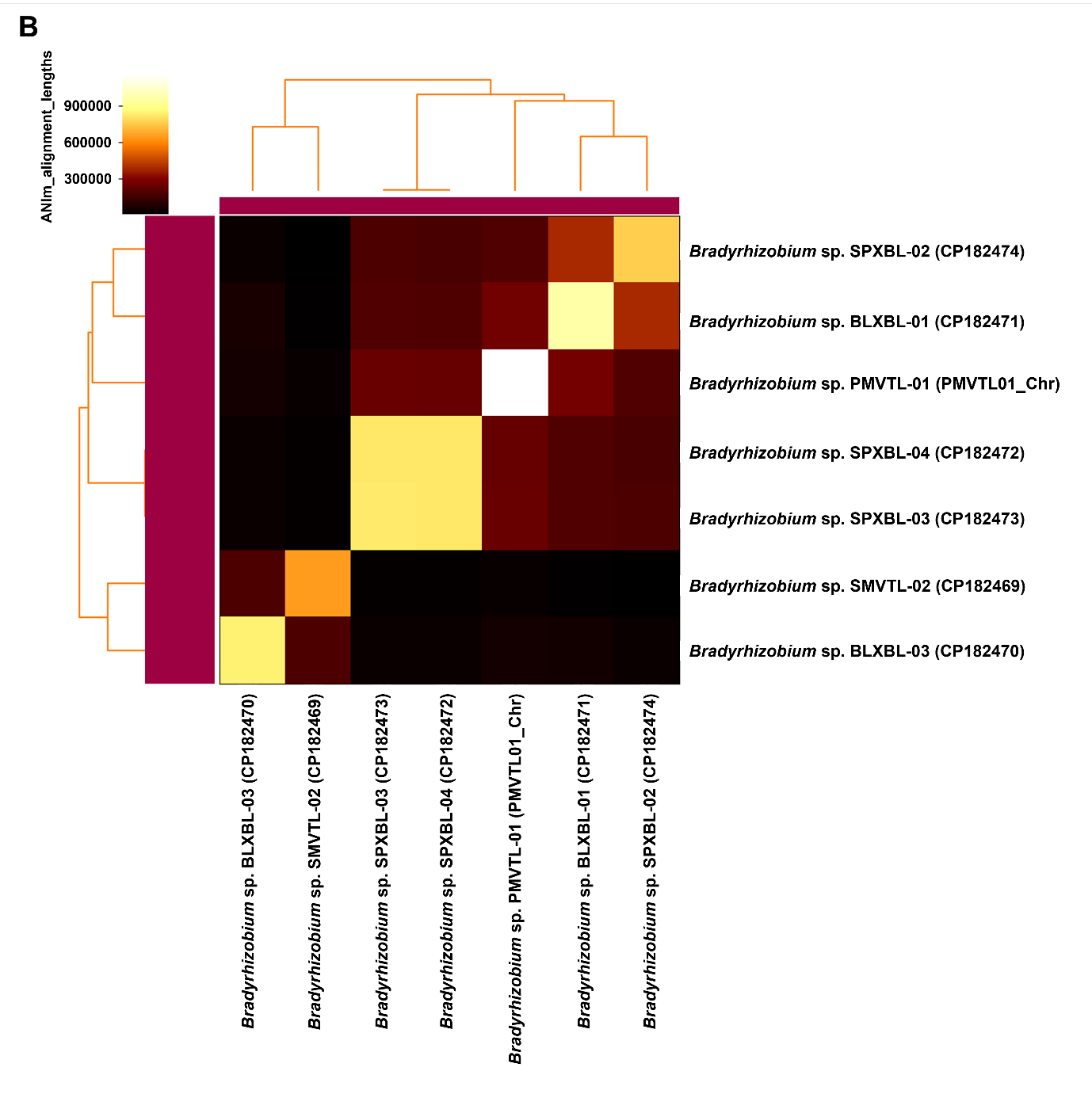


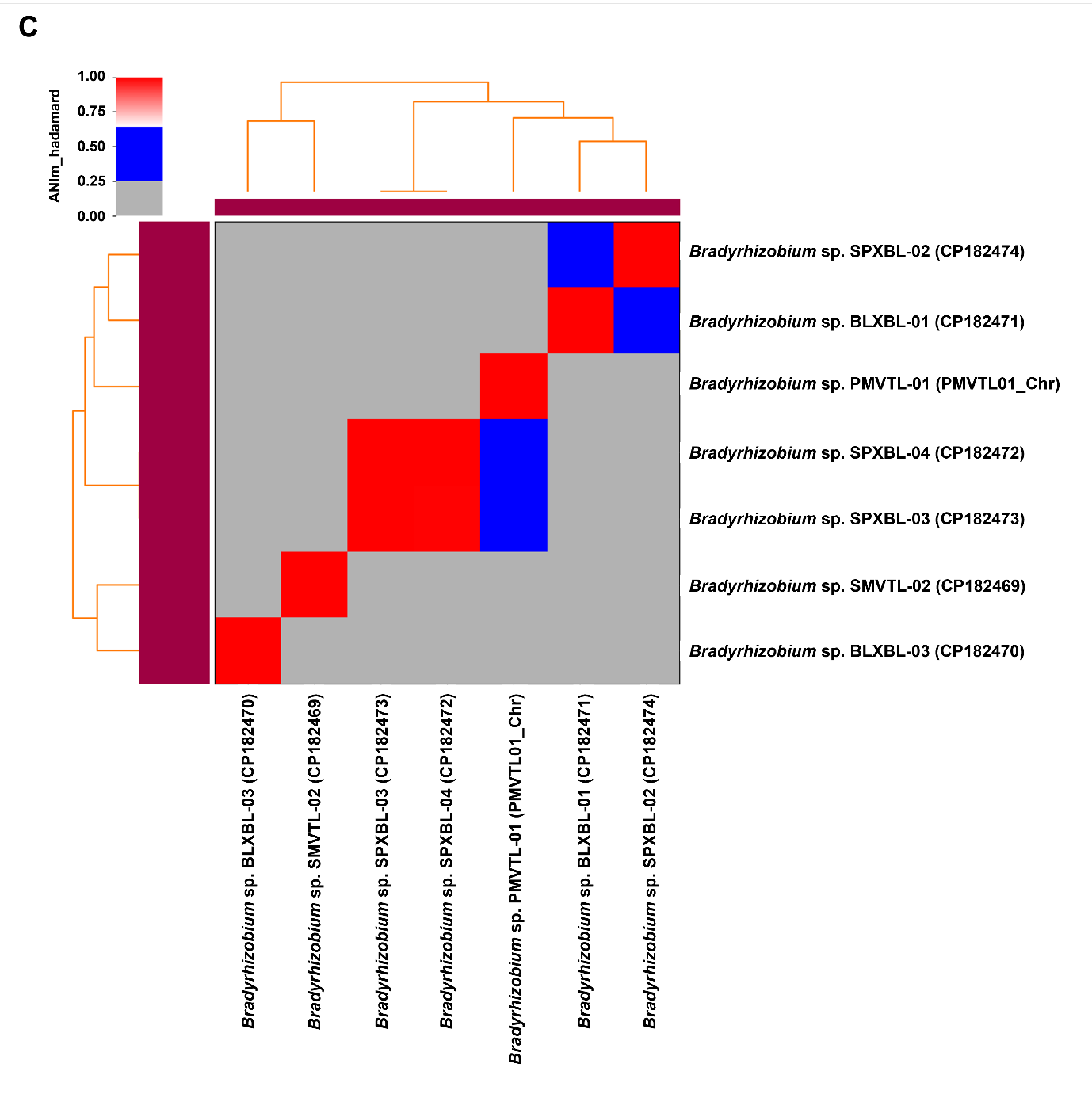


**Fig. S3** Average Nucleotide Identity (ANI) Analysis of Symbiosis Islands in *Bradyrhizobium* Strains. Heatmap representation of ANI percentage values across the symbiosis islands of seven *Bradyrhizobium* sp. strains. Red-colored cells indicate ANI values of 95% or higher, signifying high sequence conservation and potential functional similarity among symbiosis islands. In contrast, blue-colored cells represent ANI values below this threshold, suggesting significant genomic divergence. The analysis is based on five ANI metrics: **(A)** Alignment coverage, **(B)** Alignment lengths, **(C)** Hadamard transformation. These metrics provide a comprehensive assessment of sequence similarity and structural variations among the symbiosis islands.


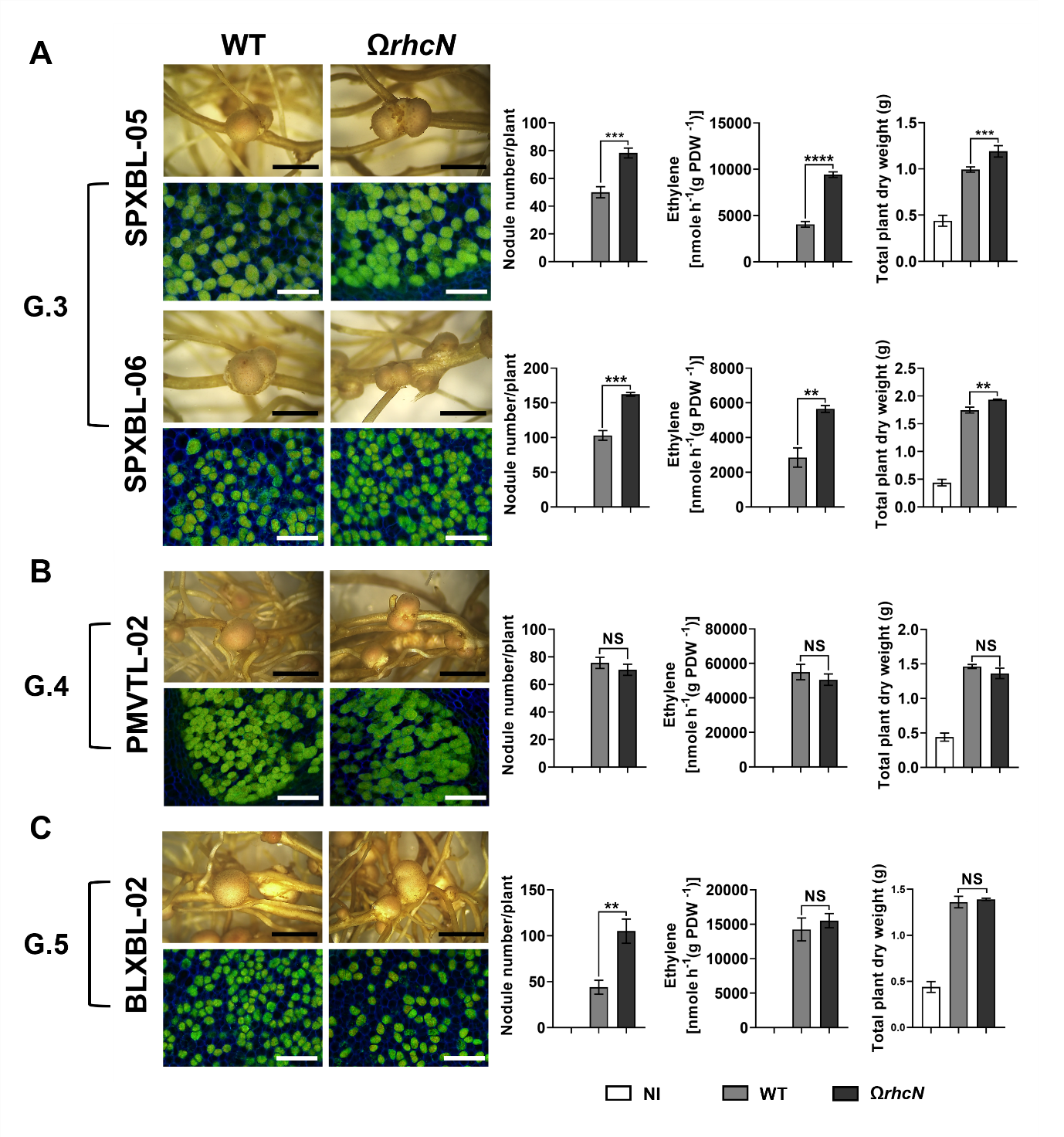


**Fig. S4** Symbiotic phenotypes of four *Bradyrhizobium* sp. strains with *Arachis hypogaea* at 30 dpi. Symbiotic phenotypes were assessed at 30 days post-inoculation (dpi) in wild-type and Ω*rhcN* mutant stains. **(A)** G3: SPXBL-05 and SPXBL-06. **(B)** G4: PMVTL-02. **(C)** G5: BLXBL-02. Bacteroides in nodules were analyzed using confocal microscopy with propidium iodide (red; infected plant nuclei, dead bacteria), SYTO9 (green; live bacteria), and calcofluor-white (blue; plant cell walls). Nodule number, nitrogen fixation (ARA), and total plant dry weight were measured at 30 dpi. Scale bars: black = 1 mm, white = 100 µm (20X). Values represent the mean ± SD (n = 3). *t*-test significance: ns = *P* > 0.05, * = *P* ≤ 0.05, ** = *P* ≤ 0.01, *** = *P* ≤ 0.001, **** = *P* ≤ 0.0001.


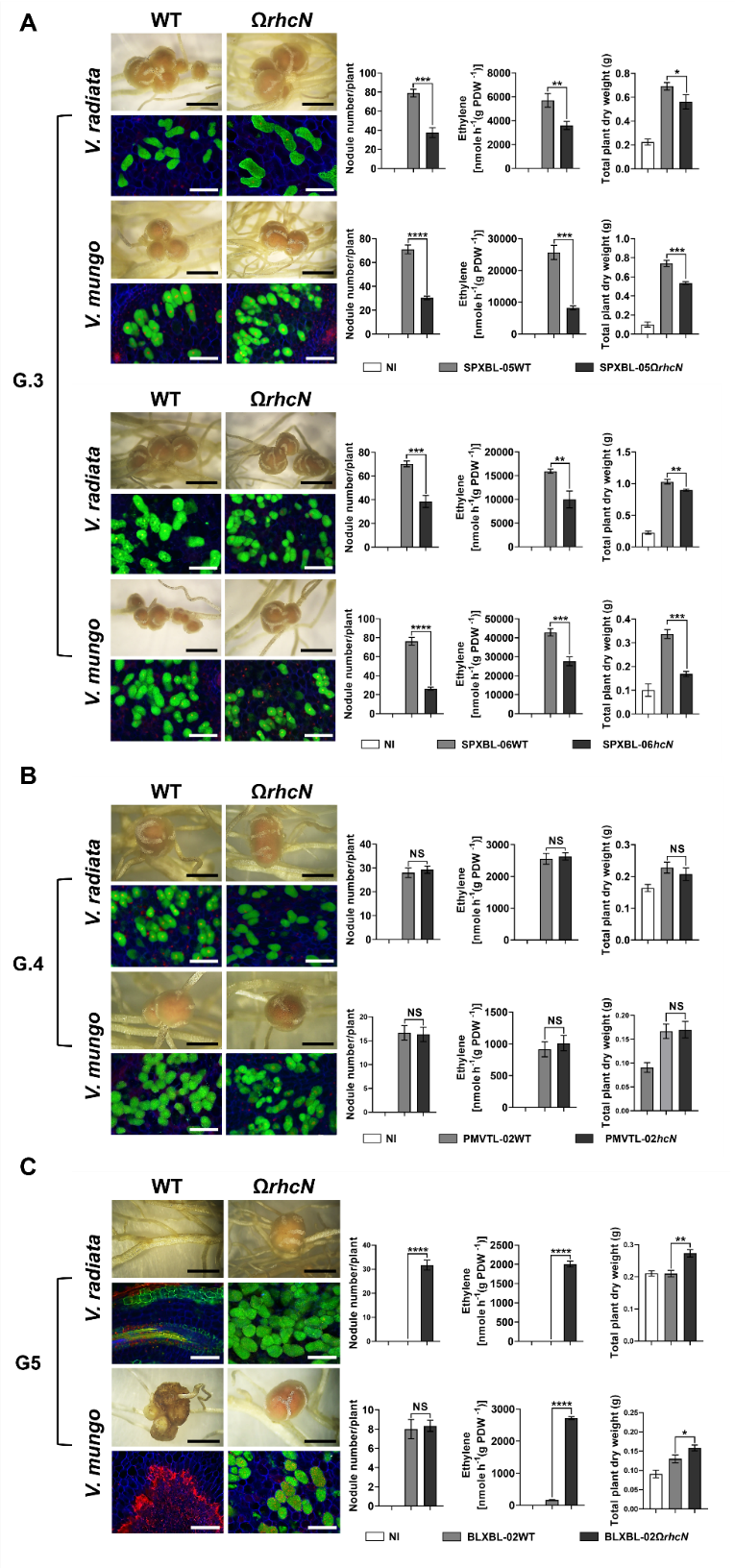


**Fig. S5** Symbiotic phenotypes of four *Bradyrhizobium* sp. strains with *V. radiata* and *V. mungo* at 30 dpi. Symbiotic phenotypes were assessed at 30 dpi in wild-type and Ω*rhcN* mutant strains. **(A)** G3: SPXBL-05 and SPXBL-06. **(B)** G4: PMVTL-02. **(C)** G5: BLXBL-02. Bacteroides in nodules were analyzed using confocal microscopy with propidium iodide (red; infected plant nuclei, dead bacteria), SYTO9 (green; live bacteria), and calcofluor-white (blue; plant cell walls). Nodule number, nitrogen fixation (ARA), and total plant dry weight were measured at 30 dpi. Scale bars: black = 1 mm, white = 100 µm (20X). Values represent the mean ± SD (n = 3). *t*-test significance: ns = *P* > 0.05, * = *P* ≤ 0.05, ** = *P* ≤ 0.01, *** = *P* ≤ 0.001, **** = *P* ≤ 0.0001.

**Table S1.** GC content of whole genome, symbiosis island and number of IS element

| **Strains** | **GC %**  **(WGS)** | **GC %**  **(Sym)** | **Length**  **(Sym)** | **Number of IS**  **(on Sym)** |
| --- | --- | --- | --- | --- |
| PMVTL-01 | 62% | 59% | 1,162,590 bp | 9 |
| SPXBL-02 | 64% | 58% | 763,635 bp | 3 |
| BLXBL-01 | 64% | 59% | 827,855 bp | 3 |
| SPXBL-03 | 63% | 59% | 821,328 bp | 5 |
| SPXBL-04 | 63% | 59% | 964,226 bp | 4 |
| SMVTL-01 | 63% | 58% | 650,751 bp | 2 |
| BLXBL-03 | 63% | 59% | 844,525 bp | 6 |

WGS, whole genome sequence; Sym, symbiotic island; Is, Insertion sequence element

**Table S2.** The bacterial strains and Plasmid

| **Strains** | **Characteristics** | **Reference** |
| --- | --- | --- |
| PMVTL-01  SPXBL-02 | Wild type strain *Bradyrhizobium* sp.  Wild type strain *Bradyrhizobium* sp. | (1)  (1) |
| BLXBL-01 | Wild type strain *Bradyrhizobium* sp. | (1) |
| SPXBL-03 | Wild type strain *Bradyrhizobium* sp. | (1) |
| SPXBL-04 | Wild type strain *Bradyrhizobium* sp. | (1) |
| SPXBL-05 | Wild type strain *Bradyrhizobium* sp. | (1) |
| SPXBL-06 | Wild type strain *Bradyrhizobium* sp. | (1) |
| SMVTL-02  PMVTL-02 | Wild type strain *Bradyrhizobium* sp.  Wild type strain *Bradyrhizobium* sp. | (1)  (1) |
| BLXBL-02 | Wild type strain *Bradyrhizobium* sp. | (1) |
| BLXBL-03  *E. coli* S17-1  *E. coli* DH5ɑ  PMVTL-01Ω*rhcN*  SPXBL-02Ω*rhcN*  BLXBL-01Ω*rhcN*  SPXBL-03Ω*rhcN*  SPXBL-04Ω*rhcN*  SPXBL-05Ω*rhcN*  SPXBL-06Ω*rhcN*  SMVTL-02Ω*rhcN*  PMVTL-02Ω*rhcN*  BLXBL-02Ω*rhcN*  BLXBL-03Ω*rhcN*  pVO155-Sm-npt2-gfp | Wild type strain *Bradyrhizobium* sp.  Contain pJQ200  *supE44 ΔlacU169 hsdR17 recA1 endA1 gyrA96 thi-1 relA1*  Insertional *rhcN* of Chr_PMVTL-01 by integration of pVO155*-Sp/Sm-npt2-gfp*; Sp/Sm^r^ at *Xba*I/*Sal*I  Insertional *rhcN* of Chr_SPXBL-02 by integration of pVO155*-Sp/Sm-npt2-gfp*; Sp/Sm^r^ at *Xba*I/*Sal*I  Insertional *rhcN* of Chr_BLXBL-01 by integration of pVO155*-Sp/Sm-npt2-gfp*; Sp/Sm^r^ at *Xba*I/*Sal*I  Insertional *rhcN* of Chr_SPXBL-03 by integration of pVO155*-Sp/Sm-npt2-gfp*; Sp/Sm^r^ at *Xba*I/*Sal*I  Insertional *rhcN* of Chr_SPXBL-04 by integration of pVO155*-Sp/Sm-npt2-gfp*; Sp/Sm^r^ at *Xba*I/*Sal*I  Insertional *rhcN* of SPXBL-05 by integration of pVO155*-Sp/Sm-npt2-gfp*; Sp/Sm^r^ at *Xba*I/*Sal*I  Insertional *rhcN* of SPXBL-06 by integration of pVO155*-Sp/Sm-npt2-gfp*; Sp/Sm^r^ at *Xba*I/*Sal*I  Insertional *rhcN* of Chr_ SMVTL-02 by integration of pVO155*-Sp/Sm-npt2-gfp*; Sp/Sm^r^ at *Xba*I/*Sal*I  Insertional *rhcN* of PMVTL-02 by integration of pVO155*-Sp/Sm-npt2-gfp*; Sp/Sm^r^ at *Xba*I/*Sal*I  Insertional *rhcN* of BLXBL-02 by integration of pVO155*-Sp/Sm-npt2-gfp*; Sp/Sm^r^ at *Xba*I/*Sal*I  Insertional *rhcN* of Chr_ BLXBL-02 by integration of pVO155*-Sp/Sm-npt2-gfp*; Sp/Sm^r^ at *Xba*I/*Sal*I  pUC119-derived suicide vector with gusA gene, GFP, and SM^r^ cassette, Km^r^ Sm^r^ Sp^r^ | (1)  (2)  Toyobo Inc.  This study  This study  This study  This study  This study  This study  This study  This study  This study  This study  (3) |

Ω, insertional mutants; *cefo*^r^, cefotaxime resistant; *Km*^r^, kanamycin resistant; *Sp/Sm*^r^, spectinomycin and Streptomycin resistant genes cassette.

**Table S3.** The details of primers and plasmid used in this study

| **Construction** | | | |
| --- | --- | --- | --- |
| **Name** | **Name of primer** | **Sequence 5’ 3’** | **Description of design (reference or accession no.)** |
| PMVTL-01Ω*rhcN*  SPXBL-02Ω*rhcN,*  BLXBL-01Ω*rhcN*  SPXBL-03Ω*rhcN*  SPXBL-05Ω*rhcN*  SPXBL-04Ω*rhcN,* SPXBL-06Ω*rhcN*  SMVTL-02Ω*rhcN*,  PMVTL-02Ω*rhcN*  BLXBL-02Ω*rhcN,*  BLXBL-03Ω*rhcN*  Universal primer  Universal primer  Universal primer  Universal primer | G1-rhcN.in.f  G1-rhcN.in.f  G2-rhcN.in.f  G2-rhcN.in.f  G3-1-rhcN.in.f  G3-1-rhcN.in.r  G3-2-rhcN.in.f  G3-2-rhcN.in.r  G4-rhcN.in.f  G4-rhcN.in.r  G5-rhcN.in.f  G5-rhcN.in.r  230-rhcN.univ.f2  231-rhcN.univ.R2  2863-pVO.Bis r  4699-CefoM15-Fin.f | ATCTTCGTCGACGAACATCGATACACGCGCTGTC  GGTCTGTCTAGAGCGACTTTTATCGGGCCCTTG  CTCCGTCGACGAGGCCGATTTCACGCATGG  GGCGTCTAGAGTCACCATCGTTGCGCTGATC  GCGCGTCGACAGCGCGTCAAAGAATCCATC  GTCGTCTAGAGTCATCGTCGTTGCGCTGATC  TCGGGTCGACTCATCGTCGTTGCGCTGATC  CACGTCTAGAGCGACTAAAGCGCGTCAAAG  TTCTGTCGACGCAAGGCCAATTTCACGCATTG  AAGGTCTAGACGCCGACGTCACTGTAGTTG  ATCCGTCGACAGAATGACGCGCAGGCCTTG  CTTATCTAGACAGATCGTAAAGGGCGCGAT  TGGTCGCTYGAGGCBGARGTNATCGGT  GCTCGCCCATRCCGGCKCGCTCCAAC  GCACAGCAATTGCCCGGCTTTCTTG  GCTATGGCACCACCAACGATATC | Designed from *rhcN* of Bradyrhizobium sp. strain PMVTL-01 (PMVTL01_Chr) this study  Designed from *rhcN* of Bradyrhizobium sp. strain SPXBL-02 (CP182474) this study  Designed from *rhcN* of Bradyrhizobium sp. strain SPXBL-03 (CP182473) this study  Designed from *rhcN* of Bradyrhizobium sp. strain SPXBL-04 (CP182472) this study  Designed from *rhcN* of Bradyrhizobium sp. strain SMVTL-02 (CP182469) this study  Designed from *rhcN* of Bradyrhizobium sp. strain BLXBL-03 (CP182470) this study  (4)  (4)  (5)  (5) |

**References**

1. Phimphong T. 2022. Selection of Bradyrhizobia for peanut production in the LAO People’s Democratic Republic. PhD Thesis. School of Biotechnology Institute of Agricultural Technology Suranaree ….

2. Giraud E, Lavergne J, Verméglio A. 2010. Chapter 9 - Characterization of Bacteriophytochromes from Photosynthetic Bacteria: Histidine Kinase Signaling Triggered by Light and Redox Sensing, p. 135–159. *In* Methods in Enzymology. Academic Press.

3. Wongdee J, Songwattana P, Nouwen N, Noisangiam R, Fardoux J, Chaintreuil C, Teaumroong N, Tittabutr P, Giraud E. 2016. nifDK Clusters Located on the Chromosome and Megaplasmid of *Bradyrhizobium* sp. Strain DOA9 Contribute Differently to Nitrogenase Activity During Symbiosis and Free-Living Growth. MPMI 29:767–773.

4. Okazaki S, Tittabutr P, Teulet A, Thouin J, Fardoux J, Chaintreuil C, Gully D, Arrighi J-F, Furuta N, Miwa H, Yasuda M, Nouwen N, Teaumroong N, Giraud E. 2016. *Rhizobium*-legume symbiosis in the absence of Nod factors: two possible scenarios with or without the T3SS. ISME J 10:64–74.

5. Piromyou P, Greetatorn T, Teamtisong K, Tittabutr P, Boonkerd N, Teaumroong N. 2017. Potential of Rice Stubble as a Reservoir of Bradyrhizobial Inoculum in Rice-Legume Crop Rotation. Appl Environ Microbiol 83:e01488-17.
